# High-power hybrid commutated converter for carbon-neutral power transmission

**DOI:** 10.1038/s41467-026-75691-5

**Published:** 2026-07-22

**Authors:** Chaoqun Xu, Zongze Wang, Zhanqing Yu, Yu Dong, Jinpeng Wu, Biao Zhao, Lu Qu, Rong Zeng

**Affiliations:** 1 Department of Electrical Engineering, Tsinghua University, Beijing, China; 2 State Grid Corporation of China, Beijing, China

**Keywords:** Electrical and electronic engineering, Energy grids and networks, Renewable energy

## Abstract

To achieve carbon neutrality, we must overcome the challenges associated with the long-distance and large-capacity transmission of renewable energy. Direct-current transmission system has advantages in terms of high voltage and high controllability over the alternating-current transmission system. First-generation line-commutated-converter-based high-voltage direct-current systems risk commutation failures that threaten the safety of power grid, whereas second-generation voltage-source-converter systems entail higher carbon emissions, lower robustness, and higher power loss. We propose a next-generation high-power hybrid commutated converter that accesses renewable power, eliminating commutation failures through its hybrid operation modes of forced and recovery-enhanced commutation, with a hybrid device connection topology. We investigate the commutation principles and present the system design. Experimental results from a 120 kV/360 MW prototype demonstrate the efficacy of this high-power converter, which has been deployed in the Lingbao super-high-voltage direct-current project in Henan, China, and Mengxi ultra-high-voltage direct-current project in Inner Mongolia, China.

## Abstract (web summary)

The study presents a high-power hybrid commutated converter system that combines forced and recovery-enhanced commutation to enable reliable, efficient long-distance transmission of renewable energy, validated at 120 kV/360 MW in real-world projects.

## Introduction

Compared with the alternating-current (AC) transmission system, direct-current (DC) transmission provides higher controllability and eliminates the capacitive charging effects inherent in AC systems. DC transmission is particularly suitable for high-voltage and large-capacity power transmission^1–4^. Globally, DC transmission underpins the integration of large-scale renewable generation into interconnected grids.

The first-generation line-commutated-converter (LCC) high-voltage direct-current (HVDC) transmission system relies on thyristor-based natural commutation. The high voltage and current ratings of thyristors enable LCC-HVDC systems to deliver large transmission capacities at low cost and low loss^5,6^. Over 30 LCC-HVDC links have been commissioned in China, including 20 ultra-HVDC cases, with a total capacity exceeding 120 GW. In 2025 alone, three additional ultra-HVDC links entered service, transmitting wind and photovoltaic power from northwestern China to central and eastern load centers. However, because thyristors are semi-controlled devices that cannot actively interrupt current, rendering LCC-HVDC systems susceptible to commutation failure^7,8^. Commutation failures occur frequently^9^, and there have been more than 70 instances in China where two or more HVDC systems failed to commutate simultaneously. In 2023, six HVDC links failed to commutate simultaneously in eastern China, causing a power deficit of 24,460 MW and a frequency drop to 49.81 Hz from 50 Hz that brought the grid to the brink of load shedding. Commutation failure thus represents a critical threat to grid security.

The second-generation voltage-source-converter (VSC) HVDC transmission system employs modular multilevel converters (MMCs) with fully controlled insulated-gate bipolar transistors (IGBTs), enabling independent control of active and reactive power and eliminating commutation failure^10^. The State Grid Corporation of China has accordingly prioritized MMC-based HVDC deployment. However, the current ratings achievable with IGBT technology constrain the per-converter transmission capacity of MMC-HVDC systems below that of their LCC-HVDC counterparts. Additionally, MMC-HVDC systems have higher carbon emissions, lower robustness, and higher power loss^11^.

Globally, the deployment of HVDC systems is being strategically advanced to address diverse regional energy challenges. Europe is focusing on cross-border interconnection and renewable integration through an emerging “super grid” based on VSC-HVDC technology, particularly from North Sea offshore wind power and southern European solar power. The US is prioritizing the resolution of internal transmission bottlenecks and enhanced grid resilience. At present, the US employs HVDC in applications ranging from back-to-back interties to long-distance transmission, supported by initiatives such as the Atlantic Offshore Wind Transmission Plan. Brazil has employed HVDC systems, including ±800 kV ultra-HVDC projects, to transmit large-scale hydropower and growing renewable resources from remote northern regions to southeastern load centers, guided by its national energy expansion plans and international collaborations for tackling future challenges such as peak shaving and smart grid integration.

China’s commitment to peak carbon emissions by 2030 and carbon neutrality by 2060 is driving a rapid expansion of renewable generation^12–17^. Building a power system with high-proportion renewable energy at its core is thus important^18,19^. By 2025, installed wind and photovoltaic capacities had reached 540 GW and 1000 GW, respectively^20–22^, with peak renewable output exceeding 520 GW^23–25^. Integrating this variable generation at scale requires a transmission infrastructure that is immune to commutation failure and compatible with carbon-neutral operation^26^.

The studies have explored the mitigation of commutation failures and enhanced power system stability. Capacitor-based topologies, such as Capacitor Commutated Converters (CCCs)^27^, Controlled Series Capacitor Converters (CSCCs)^28^, and Evolutional Line-Commutated Converters (ELCCs)^29^, insert series capacitance to sustain commutation voltage during faults, yet their immunity to commutation failure is limited, and the additional capacitors introduce new harmonic issues. Statcom and Line Commutated Converters (SLCCs) employ Static Var Generators (SVGs) connected in parallel on the AC side of the converter to support the grid by controlling the reactive power output of the SVG^30^. Controllable Line Commutated Converters (CLCCs) use additional auxiliary bridge arms to actively interrupt the current, which can resolve commutation failures; however, the substantial number of additional fully controlled devices increases cost and system complexity^31^.

The LCC-VSC hybrid topology partially mitigates commutation failure but requires two independent converter stations with separate control systems, resulting in greater complexity, a larger physical footprint, and the need for DC circuit breakers.

In this paper, we describe the reconstruction of a power conversion system that is not subject to commutation failures and propose a high-power hybrid commutated converter (HCC) for renewable energy access. The proposed HCC-HVDC system integrates the advantages of first- and second-generation HVDC systems while avoiding their respective disadvantages. The primary contribution of this work lies in the integrated presentation, development, and full-scale experimental validation of the complete HCC system. Experimental results from a 120 kV/360 MW HCC implemented in the Lingbao super-HVDC engineering project in Henan Province, China, are presented, illustrating the potential for the transmission of clean electricity. Moreover, the proposed HCC system will soon be used in the Mengxi ultra-HVDC project in Inner Mongolia, China, demonstrating readiness for carbon-neutral power transmission at scale.

## Results

### Hybrid device connection topology

**Fig. 1:**
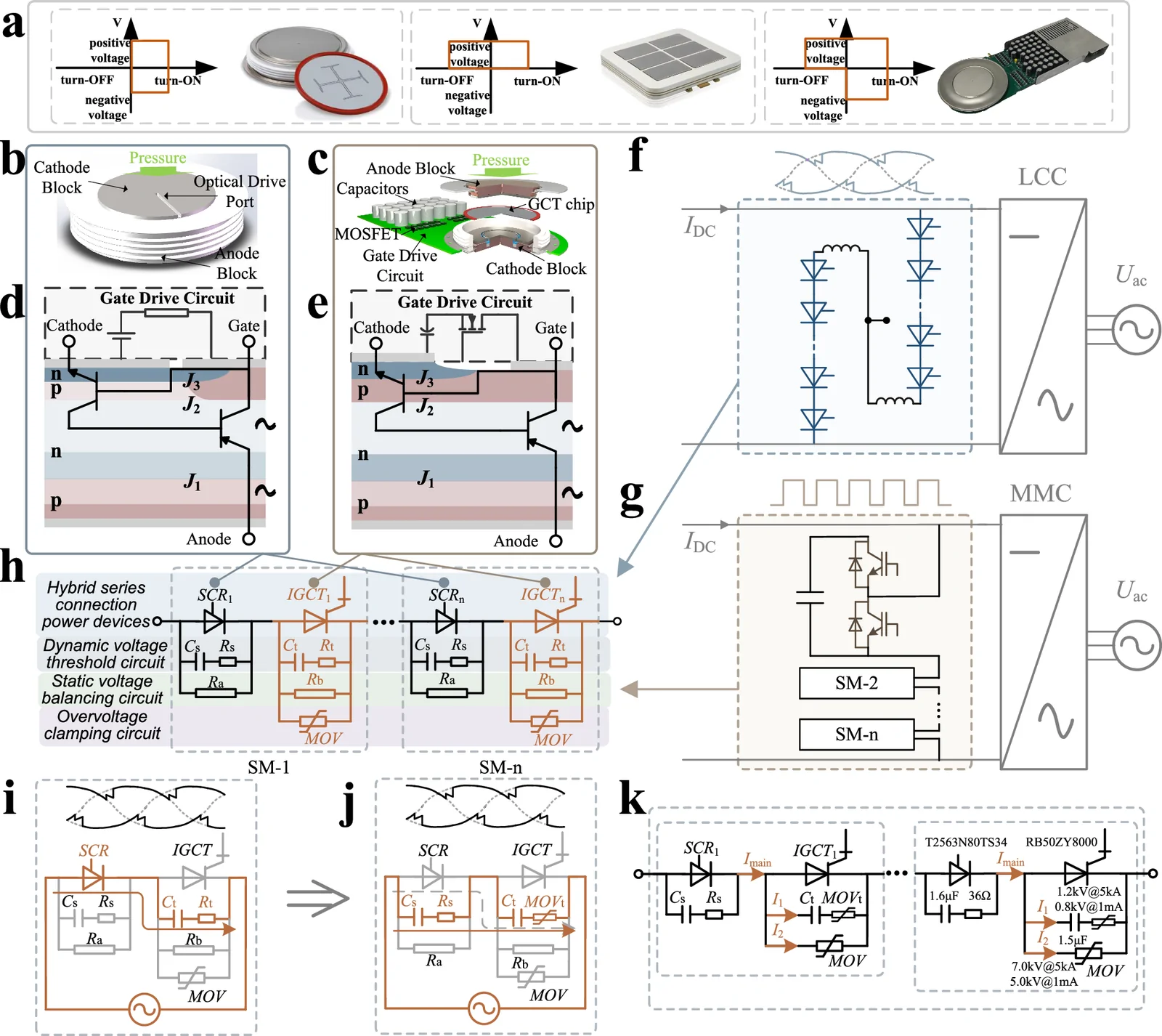
Hybrid device connection topology. **a** Operation domain comparison of the first-generation device (thyristor), second-generation device (IGBT), and new RB-IGCT device. **b** Physical schematic diagram of the thyristor. **c** Physical schematic diagram of the IGCT. **d** Cross-section of a semiconductor chip and gate drive circuit for the thyristor. *V*_on_ is the gate drive turn-ON voltage, and *R*_on_ is the gate drive turn-ON resistance. **e** Cross-section of a semiconductor chip and gate drive circuit for the IGCT. **f** Single-phase power conversion circuit of an LCC electronic system based on simple thyristors. *I*_DC_ is the DC-link current. *U*_ac_ is the AC grid voltage. **g** Single-phase power conversion circuit of the MMC electronic system based on simple IGBTs. *I*_DC_ is the DC-link current. *U*_ac_ is the AC grid voltage. SM represents the submodule. **h** Hybrid device connection topology based on hybrid thyristors and IGCTs, including a dynamic voltage threshold circuit, static voltage balancing circuit, and overvoltage clamping circuit. **i** Process of establishing the voltage at turn-OFF based on fixed resistance *R*_t_. The thyristor cannot be turned OFF owing to the latching current being exceeded. **j** Process of establishing the voltage at turn-OFF based on variable resistance *MOV*_t_. **k** Design methodology of the hybrid device connection topology.

In Fig. 1a, the thyristor lacks turn-OFF control, whereas the IGBT can only withstand unidirectional voltages by default. With the development of power semiconductor devices, the reverse blocking integrated gate-commutated thyristor (RB-IGCT) has been developed for power conversion^32,33^, by providing gate-controlled turn-OFF capability together with bidirectional voltage blocking and bridging the operating domains of thyristors and IGBTs.

The IGCT semiconductor device originated from the thyristor. A typical thyristor device comprises a chip, cathode, and anode block, as presented in Fig. 1b. A schematic diagram of the IGCT is presented in Fig. 1c; a gate drive circuit is integrated to provide a controllable turn-OFF capability. A cross-section of the thyristor chip and gate drive circuit deep inside is presented in Fig. 1d. For comparison, a cross-section of the gate-commutated thyristor (GCT) chip and gate drive circuit is presented in Fig. 1e. The physical structure and doping concentration are generally similar inside the thyristor and GCT chip. The main components of the gate drive circuit of the IGCT are the metal-oxide-semiconductor field-effect transistor (MOSFET) *T*_off_ and the energy storage capacitors *C*_off_. The thyristor and IGCT have similar turn-ON and conduction characteristics. This provides a solid theoretical basis for a hybrid series connection, but the turn-OFF transient characteristics of the thyristor and IGCT need to be regulated. Nomenclature is supplied in the Supplementary Information.

The paper proposes an HCC hybrid connection topology, as presented in Fig. 1f, g, which combines the principles of MMCs and LCCs. During stable system operation, HCC operates in a semi-controlled commutation mode inherited from the LCC, retaining large capacity, low losses, and high reliability. Under fault conditions, the fully controlled turn-OFF capability of the IGCT is activated to force-commutate the current, providing immunity to commutation failures. The series connection of semi-controlled and fully controlled devices is shown in Fig. 1h.

For hybrid series connection, the typical auxiliary circuit of the thyristor and IGCT in the HCC is designed as shown in Fig. 1h. The thyristor is paralleled by a static voltage balancing resistor with resistance *R*_a_ and a dynamic voltage threshold snubber with capacitance *C*_s_ and resistance *R*_s_; the IGCT is paralleled by a static voltage balancing resistor with resistance *R*_b_, a dynamic voltage threshold snubber with capacitance *C*_t_ and resistance *R*_t_, and an overvoltage clamping metal oxide varistor (MOV). The dynamic voltage threshold snubber limits the rate of voltage rise when the thyristor and IGCT turn OFF, and the MOV clamps the overvoltage across the IGCT.

A key design challenge is ensuring that the thyristor transitions from conduction to blocking, which requires the current to fall below the latching current. When the thyristor is turned OFF, owing to the controllable turn-OFF property of *T*_off_ in the IGCT, the AC line voltage over the IGCT generates a capacitor charging current on *C*_t_, which passes through the thyristor as shown in Fig. 1i. This current is greater than the latching current of the thyristor. The fixed resistance *R*_t_ is changed to variable resistance. In practical applications, an MOV (denoted as MOV_t_) is adopted to quickly attenuate the current to below the latching current by providing large resistance at low current to block the thyristor, as presented in Fig. 1j. During the turn-OFF transient process illustrated in Fig. 1k, the main-loop current *I*_main_ is first diverted into the dynamic voltage threshold circuit, denoted as *I*_1_. When the established voltage of the device is higher than the rated voltage of the overvoltage clamping MOV, the current transfers progressively to the MOV, denoted as *I*_2_.

The design methodology is formalized in Eq. (1); a comprehensive derivation is provided in “Methods.”

1 $$\left\{\begin{array}{c}{C}_{{{\rm{t}}}}\ge \frac{{I}_{{{\rm{main}}}}\cdot K\cdot {t}_{{{\rm{res}}}}}{{U}_{{{\rm{mov}}}}(ref)}\hfill\\ {U}_{{{\rm{mov}}}}(@{I}_{{{\rm{main}}}})\le {U}_{{{\rm{ref}}}}\hfill\\ {U}_{{{\rm{mov}}}}(ref)={U}_{{{\rm{res}}}}-{U}_{{{\rm{C}}}}({t}_{1}) > {U}_{{{\rm{res}}}}-\frac{{I}_{{{\rm{main}}}}\cdot K\cdot {t}_{{{\rm{res}}}}}{{C}_{{{\rm{t}}}}}\hfill\\ {P}_{{{\rm{mov}}}}={\int }_{0}^{{t}_{1}}{U}_{{{\rm{mov}}}}\cdot {I}_{1}\cdot {{\rm{d}}}t/t(@50{{\rm{Hz}}})\hfill\end{array}\right.$$

For the Lingbao HVDC project, rated 120 kV, 3 kA, each IGCT and thyristor has a peak blocking voltage of 8.0 kV at a nominal DC voltage of 3.4 kV, as shown in Fig. 1k. The maximum repeatable turn-OFF current of the IGCT is 5.0 kA under a 3.4 kV DC-link voltage. The residual voltage *U*_res_ and reference voltage *U*_ref_ of the MOV are designed as 7.0 kV (@5.0 kA) and 5.0 kV (@1 mA). For MOV_t_, the corresponding values are 1.2 kV (at 5.0 kA) and 0.8 kV (at 1 mA). The snubber capacitances Ct and Cs are 1.5 μF and 1.6 μF; the resistance Rs is 36 Ω.

### Forced commutation principle

**Fig. 2:**
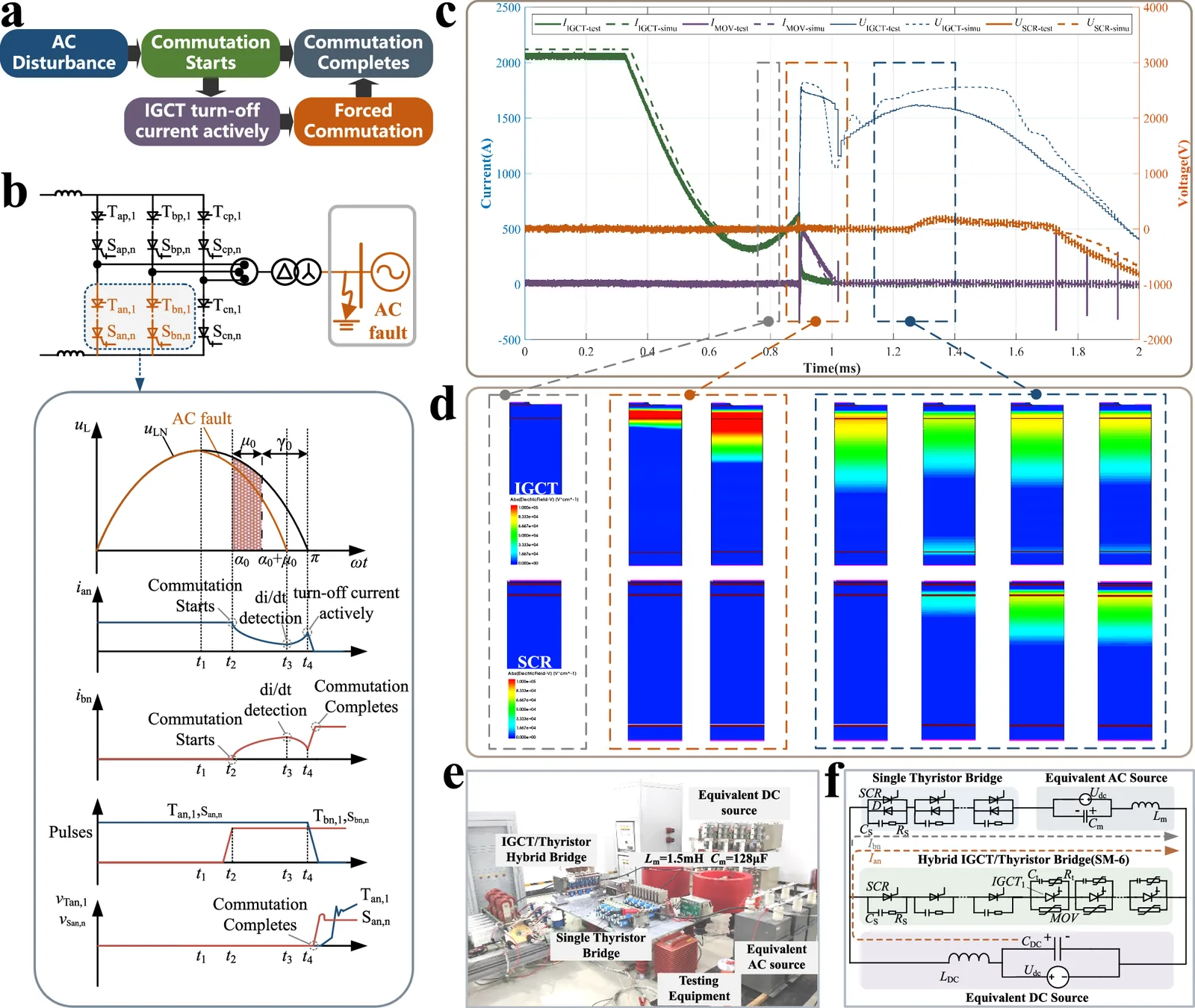
Forced commutation principle, including theoretical analysis, simulation study, and experimental study. **a** System operation strategy for forced commutation. **b** Theoretical analysis of forced commutation during the commutation process. T denotes the thyristor and S denotes the IGCT. *u*_L_ is the line AC voltage, *u*_LN_ is the rated line AC voltage, *α*_0_, *μ*_0_, and *γ*_0_ are the normal trigger angle, commutation overlap angle, and extinction angle. *i*_an_ and *i*_bn_ are the corresponding currents of phase A and phase B for the negative pole. *v*_Tan,1_ and *v*_San,n_ are the corresponding voltages of the thyristor and IGCT. **c** Consistent simulation and experimental results for the commutation process. The solid line and caption ‘test’ represent the experimental results. The dotted line and caption ‘simu’ represent the simulation results. **d** Characterization of the electric field intensity in the longitudinal section of the IGCT and thyristor chip based on finite-element simulation. The upper figures are for the IGCT, and the lower figures are for the thyristor. **e** Setup of the experimental platform for the study of the forced commutation principle. **f** Schematic diagram of the experimental circuit. The hybrid device bridge contains six submodules of six IGCTs and six thyristors.

Within the hybrid device connection, the IGCT and thyristor are combined to implement the forced commutation principle, thus eliminating commutation failures and improving the robustness and reliability of commutation. Each bridge of the HCC comprises a hybrid device connection. When an AC fault reduces the grid voltage below the level required for natural commutation, the IGCT in each bridge actively interrupts the current, forcing the commutation to complete, as shown in Fig. 2a.

The commutation process is analyzed with reference to Fig. 2b, including dynamic current commutation and dynamic voltage establishment. The current is originally scheduled to be commutated from phase A to phase B. In the schematic diagram of the HCC in Fig. 2b, the commutation proceeds from T_an,1_, S_an,n_ to T_bn,1_, S_bn,n_. When the AC fault occurs at time *t*_1_, the AC grid voltage drops from the rated value *u*_LN_, and the voltage zero-crossing point is advanced. The commutation process is assumed to start at time *t*_2_. When the line voltage *u*_L_ between phases A and B is positive, the commutation process continues, as the current of phase A, *i*_an_, is decreasing and the current of phase B, *i*_bn_, is increasing. From time *t*_2_ to *t*_3_, the thyristors and IGCTs in phases A and B both conduct current with conduction pulses. Considering the fault severity, at time *t*_3_, the AC voltage crosses zero value from positive to negative, *i*_an_ stops decreasing and rises as the fault current, and *i*_bn_ stops increasing and drops. Figure 2b shows that *i*_an_ and *i*_bn_ both have a period for which d*i*/d*t* = 0. Crucially, both currents pass through a zero-derivative interval, providing a detectable signature for triggering forced commutation. Current sensors, with a resolution of 1 A and a sampling rate of 250 kHz, can thus be installed on each bridge of the HCC for d*i*/d*t* detection. Considering the inherent oscillations and harmonics in the current waveform, our implementation incorporates a 20-sample confirmation logic. The forced commutation is triggered when d*i*/d*t* exceeds the threshold of 0.5 A/μs for 20 consecutive sampling points, actively breaking the fault current at time *t*_4_. Thus, the time period from *t*_3_ to *t*_4_ depends on the detection speed of the current sensors, which is 80 μs. With the turn-OFF action, the current is pushed from phase A to phase B. After the commutation is complete, voltages build on the thyristors and IGCTs. As the recovery of the IGCT is faster than that of the thyristor, the voltage first builds on the IGCT. After the thyristor completes its recovery, the IGCT and thyristor share the voltage establishment. Each hybrid bridge thus acts as an integrated DC circuit breaker, ensuring that commutation failure cannot occur in any commutation event.

The simulation and experimental results are in good agreement, as shown in Fig. 2c. By adopting a physics-based compact model for faster computation, the accuracy of voltage redistribution in subsequent processes was sacrificed to preserve the fidelity of the more critical active turn-OFF transient simulation. To further investigate the internal carrier concentration and the electric field of the hybrid devices, and thus verify the external electrical commutation characteristics, a finite-element simulation was conducted using Sentaurus/Technology Computer-Aided Design. The results presented in Fig. 2d show that the characteristics of the electric field for the hybrid devices are consistent with the theoretical analysis and experimental results.

The forced commutation principle was validated on a double-bridge experimental platform, as shown in Fig. 2e, f, by commutating the current from the hybrid IGCT/thyristor bridge to a single thyristor bridge under controlled fault conditions. Forced commutation thus provides immunity to commutation failure during the integration of large-capacity, variable renewable generation, enhancing the stability and robustness of the HCC-HVDC system.

### Recovery-enhanced commutation principle

**Fig. 3:**
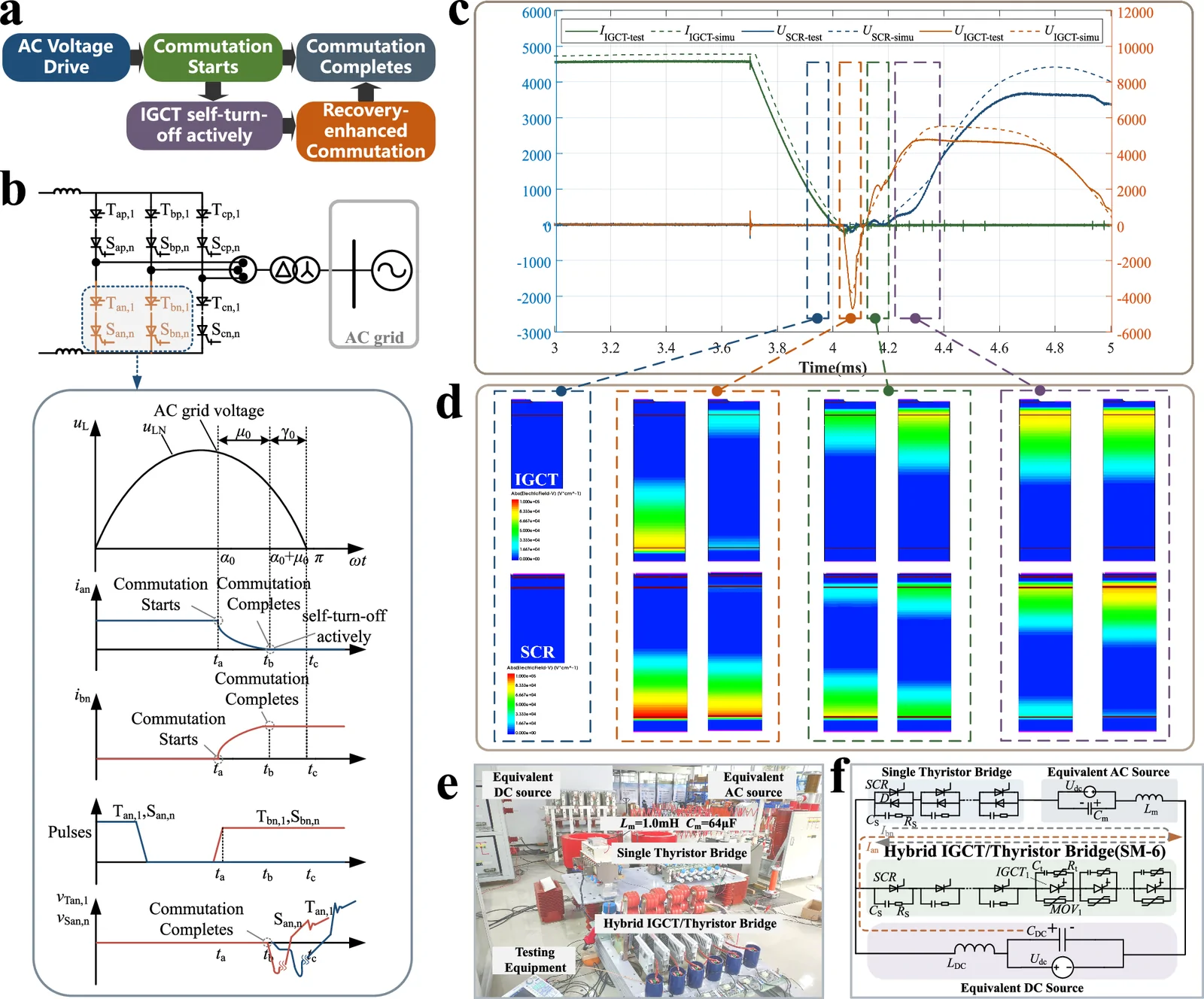
Recovery-enhanced commutation principle, including theoretical analysis, simulation study, and experimental study. **a** System operation strategy for recovery-enhanced commutation. **b** Theoretical analysis of recovery-enhanced commutation during the commutation process. **c** Consistent simulation and experiment results for the commutation process. The solid line and caption ‘test’ represent the experimental results. The dotted line and caption ‘simu’ represent the simulation results. **d** Characterization of the electric field intensity in the longitudinal section of the IGCT and thyristor chip based on finite-element simulation. The upper figures are for the IGCT, and the lower figures are for the thyristor. **e** Setup of the experimental platform for the study of the recovery-enhanced commutation principle. **f** Schematic diagram of the experimental circuit. The hybrid device bridge contains six submodules of six IGCTs and six thyristors.

In the proposed hybrid device connection, an IGCT and thyristor are combined to implement the recovery-enhanced commutation principle, achieve flexible power conversion, and improve the efficiency of commutation. When the commutation begins, if the AC grid voltage is sufficient to commutate the current, the IGCTs can self-turn-OFF the zero current actively. The operational strategy of the HCC-HVDC system for recovery-enhanced commutation is illustrated in Fig. 3a.

The recovery-enhanced commutation process is illustrated in Fig. 3b. When the current of the IGCT in phase A, *i*_an_, crosses zero at time *t*_b_, the IGCT is applied with negative voltage *v*_San,n_ by the external circuit. The negative *v*_San,n_ generates a negative current inside the IGCT. The gate drive circuit of the IGCT detects this negative voltage and negative current, and triggers a self-turn-OFF signal. The value of the negative voltage *v*_San,n_ is determined by the voltage drop characteristic of junctions *J*_1_ and *J*_2_ of the IGCT. The self-turn-OFF signal controls the MOSFET *T*_off_ in the gate drive circuit. The capacitor *C*_off_ in the gate drive circuit maintains the IGCT in the blocked state. Thus, the IGCT therefore functions as a thyristor with zero recovery time, enabling the commutation overlap angle to be reduced to zero.

The simulation and experimental results are shown in Fig. 3c. The results of finite-element simulations are presented in Fig. 3d. The characteristics of the electric field for the hybrid devices are consistent with the theoretical analysis and experimental results. In the experimental study, as shown in Fig. 3e, f, the recovery-enhanced commutation principle was validated.

The recovery-enhanced commutation principle realizes a zero-extinction angle *γ*_0_, corresponding to unity power factor operation with no reactive power consumption. The elimination of AC filters and reactive power compensation equipment reduces the station footprint and construction-phase carbon emissions by more than 30%. Thus, recovery-enhanced commutation enables efficient, filter-free HVDC operation compatible with large-scale renewable energy integration.

### Operational characteristics of the HCC system

**Fig. 4:**
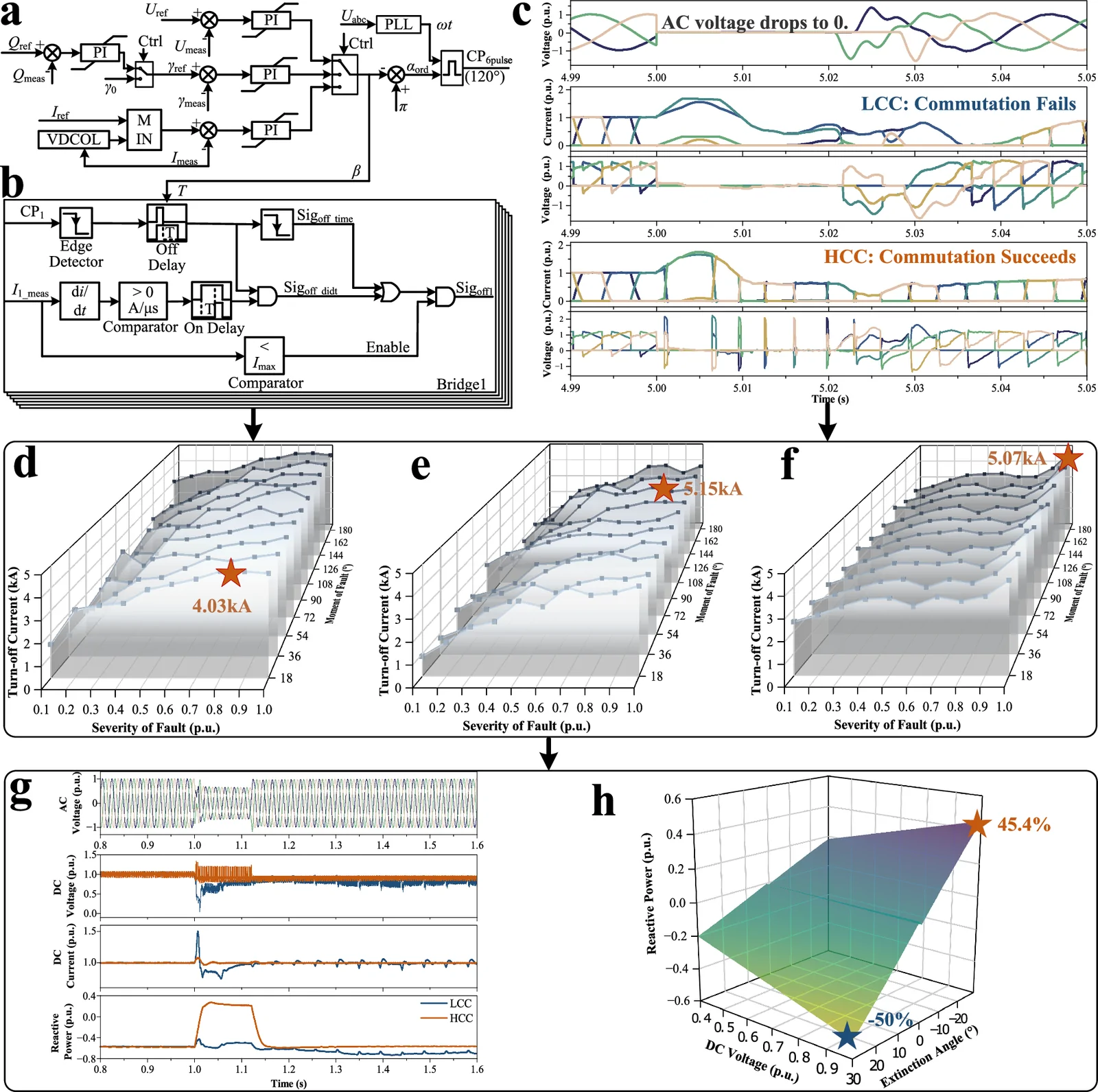
Control structure and system operation characteristics of HCC. **a** Turn-ON control structure. **b** Turn-OFF control structure. **c** Commutation of LCC and HCC during AC fault. **d** Turn-off current requirement under a single-phase ground fault. **e** Turn-off current requirement under a two-phase ground fault. **f** Turn-off current requirement under a three-phase ground fault. **g** Performance of HCC and LCC during three-phase voltage sags. **h** Reactive power support during AC voltage oscillation.

The system-level control structure of HCC consists of two parts: turn-ON control and turn-OFF control. The turn-ON control follows the conventional LCC system control structure, which includes constant voltage mode, constant current mode, and constant extinction angle mode, as shown in Fig. 4a. Generally, the rectifier adopts constant current control, while the inverter adopts constant extinction angle control. These control methods ultimately generate the nominal firing angle (*α*) required for the turn-ON signals, which are then used by a phase-locked loop (PLL) to produce six-pulse firing signals. In addition to the conventional turn-ON control, HCC introduces an additional mode for regulating the extinction angle based on reactive power demand, thereby enabling reactive power control. Specifically, two scenarios are considered regarding reactive power control. During an AC voltage dip, the reactive power reference is adjusted according to the severity of the dip—the deeper the voltage drops, the higher the reactive power reference. This predefined correspondence, in the form of characteristic curves, is stored in the control system to enable automatic reactive power adjustment in response to voltage sags. During normal operation, if the system requires reactive power support, the reactive power reference can be manually specified. The corresponding extinction angle is then calculated based on the relationship between reactive power and the extinction angle and fed into the control loop, thereby enabling the converter to provide the necessary reactive power support to the system.

The turn-OFF control is a specific control module that distinguishes HCC from LCC, with its control actions precisely targeted at each bridge arm, as shown in Fig. 4b. First, a commutation window with a duration of *β* is generated at the falling edge of the turn-ON signal, and a turn-OFF command is issued to the bridge arm at the end of this window. If a positive d*i*/d*t* is detected during the commutation window, a turn-OFF command is issued in advance at that moment to prevent further development of fault current. Furthermore, the HCC system incorporates an overcurrent protection strategy to avoid damage caused by turning off currents exceeding the capability limits of the RB-IGCT.

The system-level control structure of HCC provides effective immunity against commutation failures across all AC system fault conditions. A representative commutation failure mitigation process during a three-phase-to-ground fault is depicted in Fig. 4c. The data indicate that due to its inability to mitigate commutation failures, LCC experiences successive commutation failures after the three-phase voltage drops to zero. Once all bridge arms conduct sequentially, orderly commutation cannot be maintained, and normal operation cannot be resumed even after the AC voltage recovers. In contrast, HCC achieves orderly commutation by actively turning off the devices to construct commutation voltage. Figure 4d–f presents the maximum required turn-OFF current for successful mitigation during single-phase-, two-phase-, and three-phase-to-ground faults, respectively, under varying fault depths and initiation times. Analysis reveals that, in general, the required turn-OFF current increases with the number of faulted phases. Notably, the highest single demand for turn-OFF current, 5.15 kA, occurs under the two-phase-to-ground fault scenario. This indicates that an IGCT with a turn-OFF capability exceeding 5.15 kA enables the HCC to prevent commutation failures under all types of AC system faults.

Beyond immunity from commutation failures, the HCC exhibits a distinct advantage over the LCC in providing reactive power support to the grid during voltage sags. Figure 4g compares the performance of an LCC and an HCC under a 0.5 p.u. AC voltage dip. The HCC can flexibly absorb or supply reactive power by adjusting the extinction angle across different DC voltage levels, as shown in Fig. 4h. By adjusting the extinction angle across different DC voltage levels, the HCC can flexibly switch between absorbing and supplying reactive power. A pivotal capability is demonstrated at 1 p.u. voltage with an extinction angle of −30°, where the HCC can supply reactive power support at up to 45.4% of its nominal capacity. This performance marks a significant breakthrough for maintaining stability in critical carbon-neutral applications, such as offshore wind power transmission, where voltage regulation and system stability are essential.

### Full-scale validation of a 120 kV/360 MW HCC

**Fig. 5:**
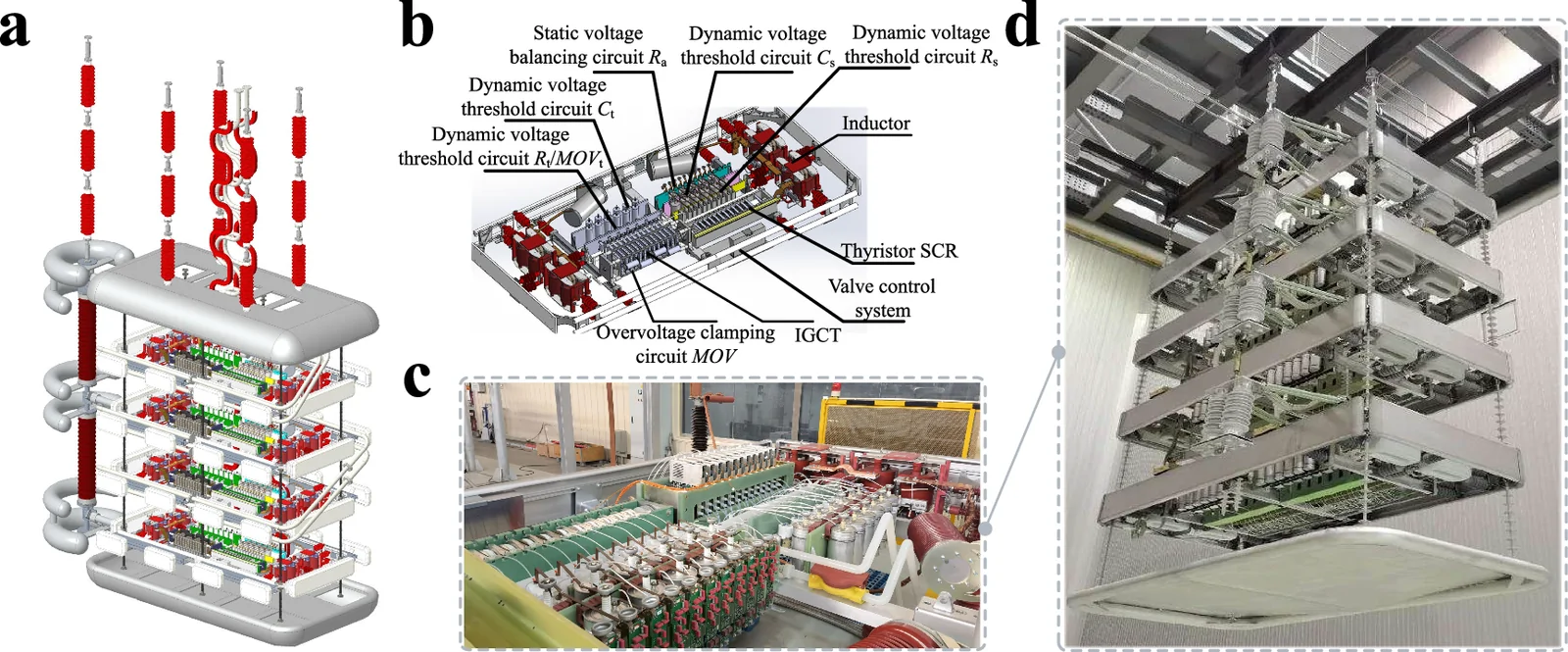
Application of a 120 kV/360 MW HCC-HVDC engineering project. **a** Physical schematic diagram of the converter valve, including four HCCs, a water-cooling system, a structure block, and a shield ring. **b** Physical module diagram of the HCC, including hybrid series-connection power devices, a dynamic voltage threshold circuit, a static voltage balancing circuit, and an overvoltage clamping circuit. **c** Physical picture of the single HCC valve. **d** Physical picture of the new HCC valve tower, which is applied in the 120 kV/360 MW Lingbao engineering project in Henan Province.

The HCC has been deployed in the 120 kV/360 MW Lingbao HVDC project in Henan Province, China, which transmits power from northwestern to central China. A schematic diagram of the HCC-HVDC structure is presented in Fig. 5a, b. The HCC valve is presented in Fig. 5c, d.

**Fig. 6:**
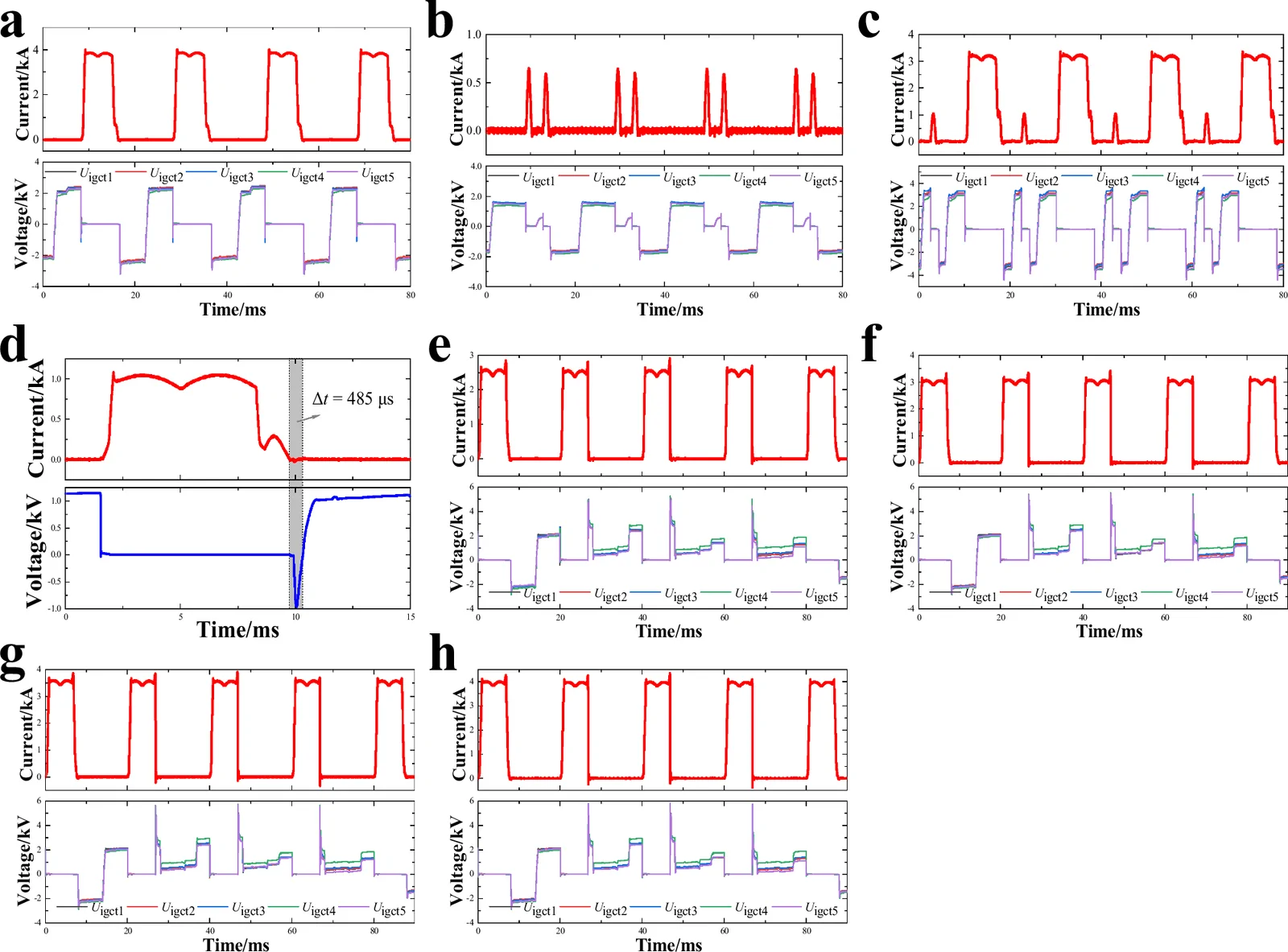
The operational performance of the HCC. **a** The experimental waveform of the maximum continuous operating load experiment. **b** The experimental waveform of the intermittent current operating experiment. **c** The experimental waveform of the maximum transient voltage operation experiment. **d** The experimental waveform of the minimum extinction angle experiment. **e** The experimental waveform of the continuous turn-OFF experiment (2500 A). **f** The experimental waveform of the continuous turn-OFF experiment (3000 A). **g** The experimental waveform of the continuous turn-OFF experiment (3500 A). **h** The experimental waveform of the continuous turn-OFF experiment (4000 A).

The operational performance of the HCC valve was characterized on a large-capacity test platform. Under maximum continuous load, the HCC sustains a current of 3780 A, as shown in Fig. 6a. During intermittent current operation (Fig. 6b), the voltage across each IGCT remains below 2 kV, decreasing to below 500 V upon re-triggering at a firing angle of 5°. Two current pulses with a width of 1.5 ms and an interval of 2.5 ms were applied. Under maximum transient voltage operation in Fig. 6c, the device-level voltage sharing remains within ±5%. The minimum extinction angle measured is 8.73°, corresponding to a current-to-voltage zero-crossing interval of 485 μs, shown in Fig. 6d. This value is substantially below the typical LCC extinction angle, confirming the viability of recovery-enhanced commutation. Figure 6e–h shows the experimental waveforms of 2500 A, 3000 A, 3500 A, and 4000 A with three consecutive wave turn-OFFs, respectively. The HCC protects against commutation failure faults in extreme situations through the forced commutation principle.

The validated HCC has been integrated into the 120 kV/360 MW Lingbao HVDC project and is further adopted for the Mengxi ultra-HVDC project in Inner Mongolia, China. The transmission line short-circuit tests of the 120 kV/360 MW HCC system were performed and succeeded, which is shown in Supplementary Video. It has demonstrated broad applications for carbon-neutral power transmission at scale.

## Discussion

**Fig. 7:**
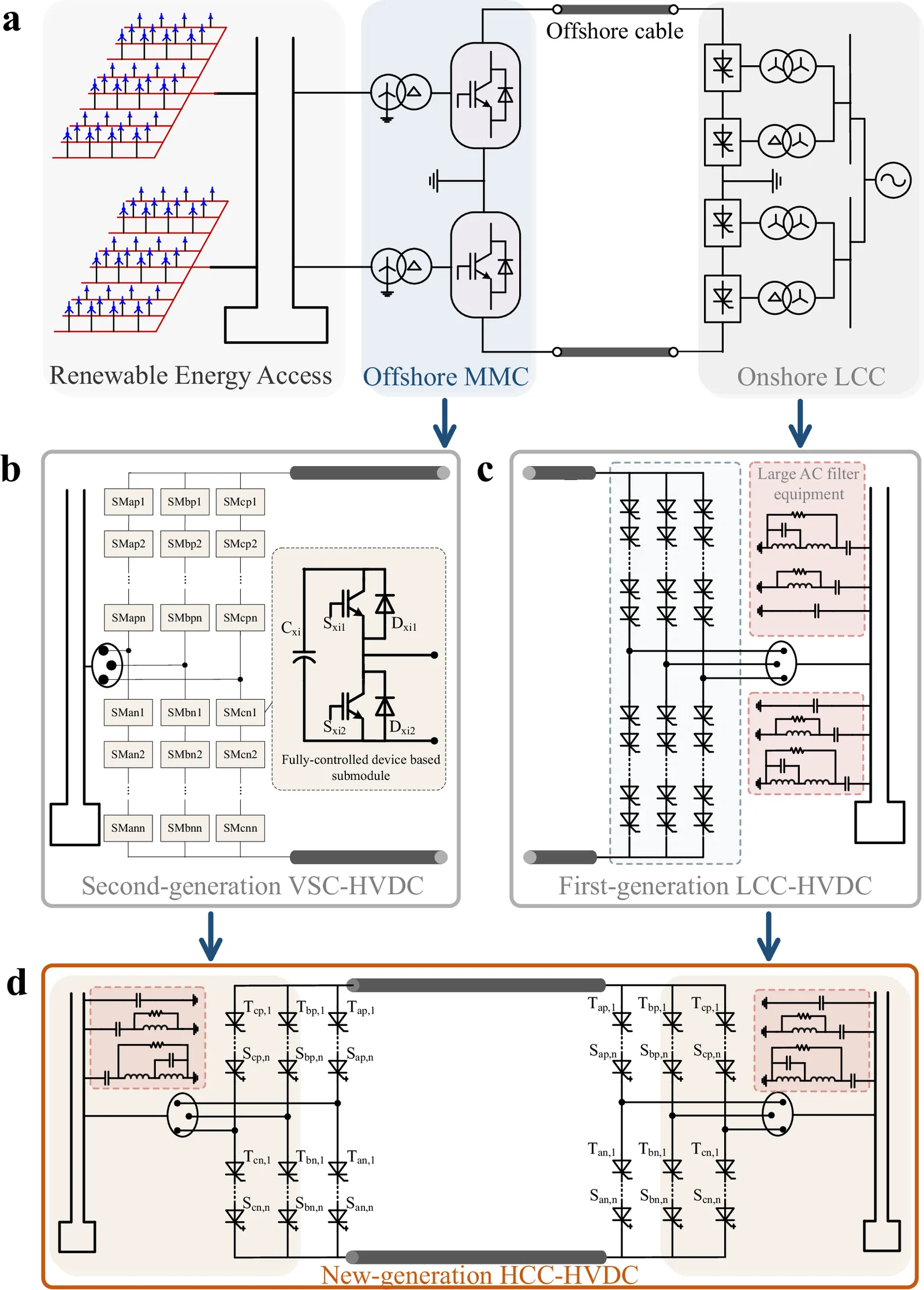
Typical scheme of applying an HCC-HVDC transmission system in accessing offshore wind power. **a** Structure of the ultra-HVDC transmission system for accessing offshore wind power, which is currently applied widely. **b** Offshore AC–DC power conversion based on the MMC. **c** Onshore DC–AC power conversion based on the LCC. **d** Offshore and onshore power conversion based on the HCC.

Large-capacity renewable-energy access is vital for carbon neutrality. To analyze the system-level HCC-HVDC scheme, access to offshore wind power energy is considered as an example, as presented in Fig. 7a. In Fig. 7b, adopting the second-generation HVDC systems, the offshore converter applies the MMC in providing an AC voltage to connect to an offshore wind farm, which results in high costs and a large physical footprint. To increase the transmission power capacity, the onshore converter applies the LCC to connect to an onshore AC power grid, as presented in Fig. 7c. However, the LCC has a commutation failure risk on the inverter side, resulting in the interruption of power transmission and an overvoltage at the sending end.

The HCC-HVDC system is proposed, which can be applied to energy access as presented in Fig. 7d. HCC employs IGCTs as its primary semiconductor device, based on the LCC architecture. As a thyristor-based technology, IGCTs not only inherit the advantages of conventional thyristors, namely high current-carrying capacity and low conduction losses, but also incorporate the active turn-OFF capability typically associated with IGBTs. When applied offshore, HCC’s dynamic voltage-support capability and wide power factor operation range are sufficient to enable the integration of renewable energy, significantly reducing both cost and physical footprint. When deployed onshore, its commutation failure immunity and fault ride-through capability help avoid interruptions in power transmission. Moreover, due to its extended operating range, HCC can utilize surplus filter banks to provide reactive power support to the grid, further strengthening its application advantages. Therefore, the proposed HCC-HVDC system effectively combines the advantages of first- and second-generation HVDC systems.

The economic argument for HCC is compelling, particularly when viewed through the lens of lifecycle cost and its role in enabling carbon neutrality. A comparative analysis is summarized in Table 1. In terms of economy, HCC incurs only a 10% cost increase compared to LCC, where a conventional ±800 kV design typically costs around 800 million CNY, whereas MMC—while also capable of resisting commutation failures and providing reactive power support—costs over three times more than LCC. Furthermore, according to actual operational data from the Lingbao converter station, the operating loss of HCC is only 0.18%, based on the rated power of the converter station, while under the same parameters, LCC shows 0.23% and MMC increases to 0.60–0.95%^34^. The critical commutation failure voltage of HCC is 0, meaning that, like MMC, HCC completely eliminates the impact of commutation failure. Additionally, unlike LCC, which requires reactive power compensation devices with 50% capacity, both HCC and MMC can deliver reactive power to the grid. Moreover, while maintaining the same control complexity as LCC, HCC distinguishes itself from the complex control of MMC^35^. In summary, the feasibility and economic advantages of using HCC for large-scale renewable energy transmission are evident.

**Table 1:** Comparison of HVDC converter technologies^a^

Aspect	LCC	VSC(MMC)	LCC-VSC hybrid^37^	HCC (This work)
Converter cost	1.0 p.u.	3.1 p.u.	2.05 p.u.	1.1 p.u.
Operational losses	0.23%	0.60–0.95%^34^	0.62%	0.18%
Commutation failure threshold voltage	0.9 p.u.	0 p.u.	0.9 p.u.	0 p.u.
Reactive power compensation cost	0.5 p.u.	0 p.u.	0 p.u.	0 p.u.
System complexity	Low	Medium^35^	Very high	Low
Key enabler for renewables	Limited by stability concerns	Excellent but costly	Effective but expensive	High-capacity, stable & cost-effective

^a^Operational and cost parameters are based on typical industry data and project reports from the State Grid Corporation of China.

For long-distance, large-scale renewable energy transmission projects, such as delivering power from remote “sand-gobi-desert” renewable bases in China or similar settings worldwide, the cost per megawatt-kilometer is a decisive factor. The economic profile of the HCC, which approaches that of LCCs while offering VSC-like stability, significantly reduces the levelized cost of electricity for clean energy. By providing a reliable, high-capacity corridor without the risk of commutation-failure-induced outages, the HCC enhances grid utilization and reduces the need for redundant backup infrastructure. This improved efficiency and reliability directly translate into lower system-wide costs and accelerated deployment of renewable energy, thereby delivering substantial carbon mitigation benefits over the project’s lifetime.

A distinguishing aspect of this work is the integrated development and validation of the complete HCC system, moving beyond theoretical concepts or isolated component studies. This encompasses the topology design, the development of key operational principles, the engineering realization of a full-scale prototype, like the 120 kV/360 MW system, and its comprehensive experimental validation. This system-level proof is crucial for transitioning from academic research to industrial applications. Nevertheless, several limitations warrant discussion. Compared with a full MMC, the HCC offers reduced topological flexibility. The current implementation operates as a grid-following converter with voltage-support capability; grid-forming operation remains an open research direction. The HCC is therefore best suited to high-capacity, point-to-point or multi-terminal transmission corridors where commutation failure immunity is the primary requirement. Future work will explore partial modularization of the HCC architecture and the development of advanced control strategies for broader grid-support functionality.

Taken together, these results establish the HCC as a viable converter technology for long-distance, large-capacity renewable energy transmission with inherent commutation failure immunity. The successful deployment at 120 kV and 360 MW, and the forthcoming adoption for ultra-HVDC transmission, indicate that the HCC architecture is ready for system-level integration into carbon-neutral power grids.

## Methods

### Design methodology of the hybrid device connection topology

When designing the parameters of the hybrid device connection topology for HCC-HVDC systems, the residual voltage of the overvoltage clamping MOV, *U*_res_, should be less than the transient withstanding voltage of the IGCT under the maximum repeatable turn-OFF current *I*_main_, for a steep-wave impulse front duration *t*_res_. The reference voltage of the MOV, *U*_ref_, should be higher than the DC-link withstanding voltage of the IGCT.

For the dynamic voltage threshold circuit, we assume that the IGCT is turned-OFF, and the time instant at which the main-loop current is fully transferred to the MOV is denoted as *t*_1_. The line inductance in the HVDC system is large, and it is thus considered that the main-loop current remains basically unchanged for a short period of time; thus, the main-loop current is approximated as constant at *I*_main_ over the turn-OFF transient. The capacitor voltage is assumed to be *U*_c_, the voltage drop of MOV_t_ is *U*_mov_, and the voltage over the IGCT is *U*. The equivalent resistance of MOV_t_ is denoted as *R*_mov_.

At any time, it holds that

2 $$U={U}_{{{\rm{C}}}}+{U}_{{{\rm{mov}}}}$$

After *t* = 0, we have

3 $$U(0)={U}_{{{\rm{C}}}}(0)+{R}_{{{\rm{mov}}}}\cdot {I}_{1}$$

4 $${R}_{{{\rm{mov}}}}(@{U}_{{{\rm{ref}}}})=\frac{{U}_{{{\rm{ref}}}}}{{I}_{1}(0)}$$

*t*_1_ is the moment when the main-loop current is fully transferred to the MOV:

5 $$U({t}_{1})={U}_{{{\rm{C}}}}({t}_{1})+{R}_{{{\rm{mov}}}}\cdot {I}_{1}={U}_{{{\rm{C}}}}({t}_{1})+{U}_{{{\rm{mov}}}}(ref)$$

6 $${U}_{{{\rm{mov}}}}(ref) > {U}_{{{\rm{C}}}}({t}_{1})$$

7 $${U}_{{{\rm{C}}}}({t}_{1})\ge {U}_{{{\rm{ref}}}}-{U}_{{{\rm{mov}}}}(@{I}_{{{\rm{main}}}})$$

It follows that

8 $$\left\{\begin{array}{c}{U}_{{{\rm{C}}}}(0)=0\hfill\\ {C}_{{{\rm{t}}}}\cdot {{\rm{d}}}U={I}_{1}\cdot {{\rm{d}}}t\hfill\\ {I}_{{{\rm{main}}}}={I}_{1}+{I}_{2}\hfill\\ U(0)={U}_{{{\rm{ref}}}}\hfill\\ U({t}_{1})={U}_{{{\rm{res}}}}\hfill\end{array}\right.$$

We therefore have

9 $${C}_{{{\rm{t}}}}\cdot {U}_{{{\rm{C}}}}({t}_{1})-{C}_{{{\rm{t}}}}\cdot {U}_{{{\rm{C}}}}(0)={\int }_{0}^{{t}_{1}}{I}_{1}\cdot {{\rm{d}}}t$$

10 $${C}_{{{\rm{t}}}}\cdot {U}_{{{\rm{C}}}}({t}_{1}) < {I}_{{{\rm{main}}}}\cdot {\int }_{0}^{{t}_{1}}{{\rm{d}}}t$$

From Equations (9)–(10), *t*_1_ is given by

11 $${t}_{1} > \frac{{C}_{{{\rm{t}}}}\cdot {U}_{{{\rm{C}}}}({t}_{1})}{{I}_{{{\rm{main}}}}}$$

The voltage rise time *t*_1_ and the time of the steep wave impulse current front *t*_res_ are set as

12 $${t}_{1}=K{t}_{{{\rm{res}}}}$$

The parameters of the dynamic voltage threshold circuit including MOV_t_ and *C*_t_ are designed as

13 $$\left\{\begin{array}{c}{C}_{{{\rm{t}}}}\ge \frac{{I}_{{{\rm{main}}}}\cdot K\cdot {t}_{{{\rm{res}}}}}{{U}_{{{\rm{mov}}}}(ref)}\hfill\\ {U}_{{{\rm{mov}}}}(@{I}_{{{\rm{main}}}})\le {U}_{{{\rm{ref}}}}\hfill\\ {U}_{{{\rm{mov}}}}(ref)={U}_{{{\rm{res}}}}-{U}_{{{\rm{C}}}}({t}_{1}) > {U}_{{{\rm{res}}}}-\frac{{I}_{{{\rm{main}}}}\cdot K\cdot {t}_{{{\rm{res}}}}}{{C}_{{{\rm{t}}}}}\hfill\\ {P}_{{{\rm{mov}}}}={\int }_{0}^{{t}_{1}}{U}_{{{\rm{mov}}}}\cdot {I}_{1}\cdot {{\rm{d}}}t/t(@50{{\rm{Hz}}})\hfill\end{array}\right.$$

Theoretical analysis of the system parameters leads to a parameter design methodology for the proposed hybrid device connection topology.

### Device-circuit coupling electromagnetic simulations

**Fig. 8:**
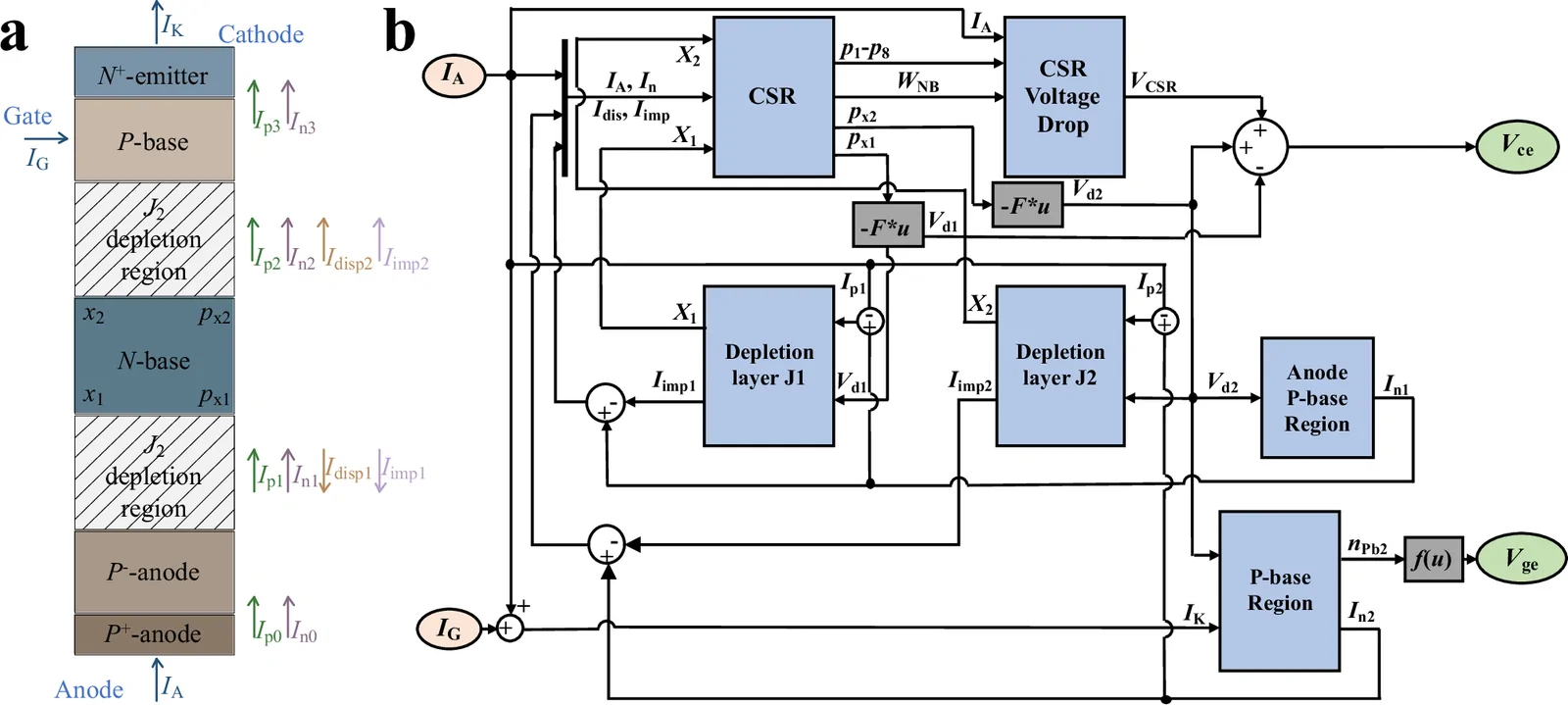
Establishment of a physics-based compact model of the RB-IGCT. **a** Establishment of seven regions of the RB-GCT chip. Current labels in the figure represent the method of transferring current between various regions. **b** Method of implementing a physics-based compact model of the RB-IGCT in MATLAB/Simulink.

A physics-based compact model of the RB-IGCT was developed to characterize the commutation dynamics of the HCC. The basic idea of the compact model is to divide the RB-GCT chip into seven regions, namely the N^+^-emitter, P base, *J*_2_ depletion layer, carrier storage region (CSR) in the N base, *J*_1_ depletion layer, anode P base, and P^+^-emitter from the cathode to the anode. There is another *J*_1_ depletion region modeled in the RB-IGCT, which results in different boundary conditions for the CSR in the N base region. The positions of the moving boundaries *x*_1_ and *x*_2_ (shown in Fig. 8a) are determined by the widths *W*_d1_ and *W*_d2_ of the depletion layers of *J*_1_ and *J*_2_. The widths and boundaries are given by Eqs. (14) and (15), where *E*_J1_ and *E*_J2_ are the electric fields within the depletion layers, *ε*_Si_ is the dielectric constant of silicon, *N*_Nb_ is the doping concentration of the N base region, *v*_sat_ is the saturation velocity, and *W*_Nb_ is the width of the N base region.

14 $$\left\{\begin{array}{c}{E}_{{{\rm{J}}}1}=\frac{{{\rm{q}}}{N}_{{{\rm{Nb}}}}}{{\varepsilon }_{{{\rm{Si}}}}}+\frac{|{I}_{{{\rm{p}}}1}|}{{\varepsilon }_{{{\rm{Si}}}}A{v}_{{{\rm{sat}}}}}-\frac{|{I}_{{{\rm{imp}}}1}|}{{\varepsilon }_{{{\rm{Si}}}}A{v}_{{{\rm{sat}}}}}\\ {W}_{{{\rm{d}}}1}=\sqrt{\frac{2{V}_{{{\rm{d}}}1}}{{E}_{{{\rm{J}}}1}}}\\ {x}_{1}={W}_{{{\rm{d}}}1}\end{array}\right.$$

15 $$\left\{\begin{array}{c}{E}_{{{\rm{J2}}}}=\frac{{{\rm{q}}}{N}_{{{\rm{Nb}}}}}{{\varepsilon }_{{{\rm{Si}}}}}+\frac{|{I}_{{{\rm{p2}}}}|}{{\varepsilon }_{{{\rm{Si}}}}A{v}_{{{\rm{sat}}}}}-\frac{|{I}_{{{\rm{imp2}}}}|}{{\varepsilon }_{{{\rm{Si}}}}A{v}_{{{\rm{sat}}}}}\\ {W}_{{{\rm{d2}}}}=\sqrt{\frac{2{V}_{{{\rm{d2}}}}}{{E}_{{{\rm{J2}}}}}}\\ {x}_{2}={W}_{{{\rm{d2}}}}\end{array}\right.$$

The voltages of the depletion layers of *J*_1_ and *J*_2_, *V*_d1_ and *V*_d2_, are derived from feedback from the boundary carrier concentrations, *p*_x1_ and *p*_x2_, as shown in Eqs. (16) and (17), where *F* is a feedback constant.

16 $${V}_{{{\rm{d}}}1}=\left\{\begin{array}{cc}0 & {p}_{{{\rm{x}}}1} > 0\\ -F{p}_{{{\rm{x}}}1} & {p}_{{{\rm{x}}}1}\le 0\end{array}\right.$$

17 $${V}_{{{\rm{d2}}}}=\left\{\begin{array}{cc}0 & {p}_{{{\rm{x2}}}} > 0\\ -F{p}_{{{\rm{x2}}}} & {p}_{{{\rm{x2}}}}\le 0\end{array}\right.$$

In contrast to the N^+^- and P^+^-emitter regions, the CSR in the N base region is considered at the high injection level, which means that the injected minority carrier concentration exceeds the majority carrier concentration. The excess carrier concentration is thus written as

18 $$\left\{\begin{array}{c}\frac{\partial p(x,t)}{\partial t}=D\frac{{\partial }^{2}p(x,t)}{\partial {x}^{2}}-\frac{p(x,t)}{{\tau }_{H}} \\ D=\frac{2{D}_{{{\rm{n}}}}{D}_{{{\rm{p}}}}}{{D}_{{{\rm{n}}}}+{D}_{{{\rm{p}}}}}\hfill \\ {\frac{\partial p}{\partial x} \Big|}_{x1}=\frac{1}{2qA} \left(\frac{{I}_{{{\rm{n}}}1} - {I}_{{{\rm{imp}}}1}} {{D}_{{{\rm{n}}}}} - \frac{{I}_{{{\rm{p}}}1}}{{D}_{{{\rm{p}}}}}\right) \\ {\frac{\partial p}{\partial x}\Big|}_{x2}=\frac{1}{2qA}\left(\frac{{I}_{{{\rm{n2}}}}-{I}_{{{\rm{imp2}}}}}{{D}_{{{\rm{n}}}}}-\frac{{I}_{{{\rm{p2}}}}}{{D}_{{{\rm{p}}}}}\right)\end{array}\right.$$

The modeling of other regions is the same as for an asymmetric IGCT^36^.

The compact model was implemented in MATLAB/Simulink, as shown in Fig. 8b, enabling device-level electromagnetic simulation of the HCC commutation process.

### Physics-based validation experiments

A hardware test platform was constructed to validate the commutation characteristics predicted by the compact model. The experimental platform is shown in Figs. 2f and 3f. *C*_DC_ comprises a 40 mF/5 kV capacitor bank. The corresponding inductance values of the three *L*_DC_ taps are 0.6, 1.0, and 1.5 mH. *U*_DC_ is the charging source, with a maximum charging current of 1 A. *C*_s_ and *R*_s_ are 1.0 μF and 25 Ω, respectively. *C*_t_ is 1.0 μF. *R*_t_ comprises *MOV*_t_, whose residual voltage and reference voltage are 1.2 kV (@5.0 kA) and 0.6 kV (@1 mA), respectively. The residual voltage and reference voltage of the MOV are 3.6 kV (@5.0 kA) and 2.4 kV (@1 mA), respectively.

## Supplementary information

Supplementary Information

Description of Additional Supplementary Files

Supplementary Video

Supplementary Code

Transparent Peer Review file

## Source data

Source Data

## Data Availability

All data that support the findings of this study are available within this article. Source data are provided with this paper.

## Code availability

The device-circuit coupling electromagnetic simulations of HCC in the study were performed in MATLAB/Simulink to characterize the commutation dynamics of the HCC. It includes an equivalent DC source, a hybrid bridge, a thyristor bridge, and an equivalent AC source. The IGCT in the hybrid bridge adopts a physics-based compact model. The code is provided with this paper.
