# Supplementary material for: High-power hybrid commutated converter for carbon-neutral power transmission
Source: Nat Commun. 2026 Jul 22;17:8912. doi: 10.1038/s41467-026-75691-5 (PMC13503722; doi:10.1038/s41467-026-75691-5)
Supplement: Supplementary file 1 [file 41467_2026_75691_MOESM1_ESM.pdf]

Supplementary Information for

High-power Hybrid Commutated Converter for Carbon-Neutral Power Transmission

Chaoqun Xu[1,2,†], Zongze Wang[1,†], Zhanqing Yu[1*], Yu Dong[2], Jinpeng Wu[1], Biao Zhao[1], Lu Qu[1] & Rong Zeng[1*]

[1]Department of Electrical Engineering, Tsinghua University, 100084 Beijing, China.

[2]State Grid Corporation of China, 100031 Beijing, China.

[†]These authors contributed equally: Chaoqun Xu, Zongze Wang.

[*]Correspondence should be addressed to Zhanqing Yu (email: yzq@tsinghua.edu.cn) or Rong Zeng (email: zengrong@tsinghua.edu.cn)

This PDF file includes nomenclature:

R_a, R_b	Static voltage balancing resistance (Ω)
C_s, C_t	Dynamic voltage snubber capacitance (μF)
R_s, R_t	Dynamic voltage threshold snubber resistance (Ω)
R_{mov}	Equivalent resistance of MOV_t (Ω)
I_{main}	Main-loop current (A)
I_1	Current of dynamic voltage threshold circuit (A)
I_2	Current of overvoltage clamping MOV (A)
U	Voltage over the IGCT (V)
U_C	Voltage over snubber capacitor (V)
U_{mov}	Voltage of MOV_t (V)
U_{ref}	Reference voltage of MOV (V)
U_{res}	Residual voltage of MOV (V)

t_1	Time of voltage rising to U_{res} (s)
t_{res}	Time of steep wave impulse current front (s)
K	Nonlinear coefficient of MOV
P_{mov}	Designed rated power of MOV (W)
E_{J1}, E_{J2}	Electric fields within the depletion layers (V/cm)
N_{Nb}	Doping concentration of the N base region (cm^{-3})
ε_{Si}	Dielectric constant of silicon (F/cm^2)
W_{d1}, W_{d2}	Depletion layers of J_1 and J_2 (cm)
v_{sat}	Saturation velocity (cm/s)
W_{Nb}	Width of the N base region (cm)
p_{x1}, p_{x2}	Boundary carrier concentrations (cm^{-3})
F	Feedback constant ($V\cdot cm^3$)
V_{d1}, V_{d2}	Voltages of depletion layers (V)
A	Total active area (cm^2)
q	Electric charge (C)
D	Ambipolar diffusivity (cm^2/s)
D_n	Electron diffusivity (cm^2/s)
D_p	Hole diffusivity (cm^2/s)
I_{n1}, I_{p1}	Electron and hole current at x_1 edge of undepleted drift region (A)
I_{n2}, I_{p2}	Electron and hole current at x_2 edge of undepleted drift region (A)
τ_H	High-level lifetime (μs)
