# Supplementary material for: High-power hybrid commutated converter for carbon-neutral power transmission
Source: Nat Commun. 2026 Jul 22;17:8912. doi: 10.1038/s41467-026-75691-5 (PMC13503722; doi:10.1038/s41467-026-75691-5)
Supplement: Supplementary file 2 [file 41467_2026_75691_MOESM2_ESM.pdf]

Description of Supplementary Files for

High-power Hybrid Commutated Converter for Carbon-Neutral Power Transmission

File Name: Supplementary Video

Description: The video of the alternating current transmission line short-circuit tests of the 120 kV/360 MW Lingbao HCC system in Henan, China. The video demonstrates that artificial short-circuit grounding faults are applied on the transmission lines to verify the performance of the 120 kV/360 MW HCC system.

File Name: Supplementary Code

Legend: The device-circuit coupling electromagnetic simulations of HCC in the study were performed in MATLAB/Simulink to characterize the commutation dynamics of the HCC. It includes an equivalent DC source, a hybrid bridge, a thyristor bridge, and an equivalent AC source. The IGCT in the hybrid bridge adopts a physics-based compact model. The code is provided with this paper.
