# Supplementary material for: High-power hybrid commutated converter for carbon-neutral power transmission
Source: Nat Commun. 2026 Jul 22;17:8912. doi: 10.1038/s41467-026-75691-5 (PMC13503722; doi:10.1038/s41467-026-75691-5)
Supplement: Supplementary file 3 [file 41467_2026_75691_MOESM5_ESM.pdf]

nature portfolio

Peer Review File

High-power Hybrid Commutated Converter for Carbon-Neutral Power Transmission

Corresponding Author: Professor Zhanqing Yu

This file contains all reviewer reports in order by version, followed by all author rebuttals in order by version.

Version 0:

Reviewer comments:

Reviewer #1

(Remarks to the Author)
The paper proposes a novel hybrid HVDC converter topology that integrates both IGCTs and thyristors. The methodology is thoroughly validated through experiments and simulation studies, and the evaluation has been carried out at a sufficiently practical scale. The paper is well-structured, and the potential impact of the proposed topology appears to be significant. My only major concern relates to the economic assessment of the proposed solution. Detailed comments are as follows:

The proposed topology should be benchmarked against conventional alternatives through an economic-benefit analysis to clearly demonstrate its cost-effectiveness.

In particular, comparison with the most widely adopted hybrid HVDC configuration (LCC on the grid side and MMC on the offshore side) is essential. The advantages of the proposed approach, especially in terms of economic performance, should be explicitly discussed. I recommend large table to compare.

(Remarks on code availability)

Reviewer #2

(Remarks to the Author)
This paper proposes a High-Power hybrid-commutated converter (HCC) HVDC system to address the commutation failure issue in conventional LCC-HVDC systems through the hybrid operation modes of forced commutation and recovery-enhanced commutation. Both simulation and test results have been shown in this paper and this HCC technology has been applied in a real HVDC project in China. All of these demonstrate that it is practical research. However, this work should be compared with existing works to highlight its contribution and novelty.

Questions are presented below:

1. Abstract (P1, Line 5): the statement 'however, they still face the risk of commutation failure, threatening the safety of the power grid' is true in the LCC-HVDC system and this issue has been widely discussed in the literature. However, it does not apply to VSC-HVDC systems. The author should clarify this to avoid unnecessary confusion.

2. Section following the abstract (P1) authors introduce the HVDC development in China, the impact of commutation failure in the LCC system, limitation of MMC-HVDC. However, there is no comparison with existing works? Has any research been done in this area? If so, please review existing methods and highlight the contribution/novelty of this paper.

3. Result A (P3, Line 23 to 26): authors propose that the HCC combines both MMCs and LCCs, thus leveraging the advantages of MMCs and LCCs. Please specify what advantages of MMCs and LCCs are being used. In addition, as it uses a hybrid structure, are there any limitations or trade-offs compared to the pure LCC or MMC system? This clarification could help readers understand the proposed technology.

4. Result B (P4, Line 17-18: the di/dt of current on each bridge was used to trigger the forced commutation principle.

Theoretically, it should use the point where di/dt=0. However, considering the disturbances in real projects like noises, a threshold of 0 might lead to the maloperation of the forced commutation as it is too sensitive. Please clarify, 1) how to set the threshold of di/dt in real implementation? What factors should be considered during the threshold selection?

5. More context should be provided to explain the results in Fig 2.C. For example, why is a larger discrepancy between the test and simulation results observed on the blue curve?

6. Discussion (P7, Line 75-83): this part states that the HCC-HVDC system is effective in long-distance and large-scale renewable energy transmission. Why in this paper a 120kV/360 MW HVDC link used as an example? The voltage and power in this link are not too high compared to the UHVDC (≥800 kV), as mentioned by the author.

7. The arrows in Figure 5 should be plotted using professional plotting tools, rather than the hand-drawn.

(Remarks on code availability)

Reviewer #3

(Remarks to the Author)
The topic of this paper, dealing with relatively new HVDC converter technology, is both relevant and timely, given the growing importance of HVDC systems. The authors have described the technology, and the presented results in the manuscript can be demonstrated by attached materials. Nevertheless, this reviewer identifies several critical points that should be addressed to further strengthen the contribution.

1. Scope of literature review
The current manuscript appears to focus mainly on the status of HVDC deployment in a specific country. While it is understandable that most HVDC applications have been concentrated there, a broader overview covering worldwide developments would be highly beneficial for the readers. For instance, European projects often provide publicly available information that could help balance the review. Since many readers and reviewers may not have access to the same depth of information from that specific country as much as authors can do, a more global perspective would enhance the completeness of the manuscript.

2. Clarification of novelty
The novelty of the paper is not sufficiently clear. Is the manuscript intended as a review paper? If so, it should more closely follow the structure and depth of a review article, including a comprehensive overview of the current state of the field. Alternatively, if the novelty lies in introducing a new generation of HVDC converters (such as HCC), then this needs to be clearly articulated. As it stands, the paper leaves some ambiguity, particularly since there already exist numerous publications on the same topic, including from the authors.

3. Literature review on HCC and related technologies
Closely related to the previous point, the manuscript currently lacks a thorough literature review of existing work on HCC. Given that many relevant publications are available, including from the authors themselves, it is important to situate the present work within that context. Furthermore, as the authors emphasize HCC's high-capacity potential, it would be valuable to review previous efforts addressing comparable challenges, such as hybrid LCC–VSC HVDC systems and bipole HVDC configurations.

4. Operational aspects beyond device-level characterization
The current manuscript mainly discusses HCC from a component-level characteristics, but provides limited insight into its operational behavior. This aspect is really important to be attractive for the application to replace with currently available one such as LCC and VSC types. Therefore, fair comparison with LCC and VSC in terms of power system operational aspect is also should be highlighted otherwise, it is not attractive as authors insisted.
The reviewer suggests that including basic operational characteristics would strengthen the work. For example, an illustration of the basic control structures, dynamic responses to power reference changes. In addition, comparisons with LCC and VSC technologies—highlighting controllability, reactive power requirements, and operational flexibility—would better demonstrate HCC's advantages. E.g., a discussion of reactive power consumption would be highly informative for the comparison with LCC. In short, reactive power characteristics compared to LCC & superior controllability for the active & reactive power compared to VSC.

5. Clarification of HCC illustration
In Figure 5(d), HCC is illustrated as a combination of SCR and IGBT. This raises questions regarding how higher capacity is achieved in practice, as the same current would flow through the IGBT as much as flow through SCR. It would be helpful for the authors to clarify whether the figure accurately reflects the intended structure, or whether it may have been oversimplified or misinterpreted. If the figure is correct, the illustrated HCC, it seems improved LCC rather than VSC by improved

commutation characteristics. Can the authors elaborate further on this aspect?

6. Grid-forming and system-level features
The manuscript suggests that HCC could enable offshore wind integration, as implied in Figure 5(d). However, the power system–level implications remain unclear. Specifically, it would be important to discuss whether and how HCC can provide grid-forming (GFM) capabilities, and to demonstrate its active/reactive power performance in a system-level context. Such analysis would complement the device-level discussion and underscore the practical value of the proposed technology. (e.g., LCC HVDC can not provide this function. How about HCC?).

(Remarks on code availability)

Reviewer #4

(Remarks to the Author)
Comments:
The paper introduces an interesting concept with practical applications; several aspects could benefit from further clarification and deeper analysis to enhance its technical rigor and impact.
1. The structure and flow of the paper could be improved for readability. For example, the results section should not appear right after the introduction; please follow the section order as per the guidelines.
2. The paper highlights novelty in hybrid operation modes and device connection topology. To strengthen this claim, could you add a clearer comparison with existing hybrid HVDC concepts (e.g., LCC/VSC hybrids, series/parallel topologies)? It would also help if you explained which limitations of those approaches the proposed HCC addresses for clear understanding of the readers.
3. The paper introduces ‘forced commutation’ and ‘recovery-enhanced commutation.’ To improve clarity, it would help to include a more detailed mathematical framework or flowcharts showing their operation under different fault conditions and to explain more clearly how the two modes differ.
4. The mathematical equations' variables should be clearly defined to easily grasp the idea.
5. The paper mentions carbon neutrality and reduced footprint. It would be helpful to include an economic analysis comparing HCC-HVDC with LCC- and VSC-HVDC and to quantify the carbon benefits with clear metrics.
6. Improve the English and correct minor grammatical errors and formatting inconsistencies.
7. Do elaborate on the limitations and potential improvements of the proposed HCC method.
By addressing these points, the paper can improve its clarity, making it more impactful and accessible to the reader.

(Remarks on code availability)
I only revised the manuscript and provided comments for further improvement. I did not review or test the code, so I cannot comment on its reproducibility or usability.

Version 1:

Reviewer comments:

Reviewer #2

(Remarks to the Author)
Thank you for the authors’ response and revisions. All comments have been properly addressed, and the reviewer has no further comments.

(Remarks on code availability)

Reviewer #3

(Remarks to the Author)
The reviewer appreciates the authors’ previous responses. This reviewer still has several key concerns of HCC for power transmission system as this paper suggested.

- Figure 6 should be revised for clarity. As noted in the authors’ response, an HCC-based HVDC system cannot access offshore renewable energy sources without grid-forming resources, either from generators or converters. However, the current representation of Fig. 6 may suggest that the HCC can directly interface with offshore renewables, which introduces ambiguity. In particular, since the offshore MMC is replaced by the HCC, Fig. 6(d) may be misleading and should be clarified or modified accordingly.

- In addition, it is unclear why no harmonic filter is shown for the HCC–HVDC system. Does the HCC exhibit harmonic performance comparable to that of an MMC, or would a harmonic filter still be required? Elaborating on this aspect will be beneficial for power system community.

- In Figure 4, the AC voltage waveform is not shown. Including the AC voltage signal, in time domain rather than static plotting as currently does, would improve clarity. Furthermore, the manuscript does not clearly explain how reactive power is controlled during AC voltage interruption. The control mechanisms and schemes should be explicitly described. The authors are encouraged to provide time-domain results for a three-phase fault case as well to highlight its reactive power support capability as it is one of main advantaes of HCC HVDC compared to LCC. It would also be helpful to clarify whether the HCC can provide reactive power support under normal operating conditions.

- As pointed out in the reviewer's previous comments and in relation to the above issues, the system-wide control structure of the HCC–HVDC system should be provided. Providing the corresponding control structure would significantly improve clarity. This should include system-wide control structures such as the outer control loops (Power & Reactive power), current controllers, and the synchronization mechanism (e.g., PLL or alternative methods). Otherwise, it is remained unclear how it works for transmission system.

(Remarks on code availability)

Reviewer #4

(Remarks to the Author)
The author has thoroughly answered questions in the revised manuscript. However, to improve the content, it needs to be arranged the format according to the journal guidelines.

1. The manuscript does not fully follow the journal's sectioning guidelines. Specifically, the Introduction section is not clearly labeled after the Abstract, and the Nomenclature section is also missing an explicit heading. This lack of proper section titles may cause confusion for readers. Please revise the manuscript to ensure that all sections are clearly titled and structured in accordance with the journal's formatting guidelines.

2. The overall quality of English needs improvement. The authors are advised to revise the manuscript carefully and consider professional language editing to enhance clarity, grammar, and readability.

(Remarks on code availability)

Version 2:

Reviewer comments:

Reviewer #3

(Remarks to the Author)
The authors addressed the comments.

(Remarks on code availability)
N/A

Open Access This Peer Review File is licensed under a Creative Commons Attribution 4.0 International License, which permits use, sharing, adaptation, distribution and reproduction in any medium or format, as long as you give appropriate credit to the original author(s) and the source, provide a link to the Creative Commons license, and indicate if changes were made.
In cases where reviewers are anonymous, credit should be given to 'Anonymous Referee' and the source.
The images or other third party material in this Peer Review File are included in the article's Creative Commons license, unless indicated otherwise in a credit line to the material. If material is not included in the article's Creative Commons license and your intended use is not permitted by statutory regulation or exceeds the permitted use, you will need to obtain permission directly from the copyright holder.
To view a copy of this license, visit https://creativecommons.org/licenses/by/4.0/

Response Letter (NCOMMS-25-43025A)

Dear Editors, Associate Editors and Reviewers,

We are very grateful to you and the reviewers for his/her appreciation of this work and the time invested in reviewing the original manuscript (ID: **NCOMMS-25-43025-T**). We have amended the relevant parts in the manuscript according to the suggestions, which are all denoted by the red font. All of the questions were answered below item by item in this letter.

For ease of referencing, the reviewers' comments are reproduced in ***black*** and our responses are given in ***blue***. The revised portions are marked in ***red*** in the new manuscript and the response letter.

In the manuscript, we propose a new-generation high-power hybrid-commutated-converter (HCC) high-voltage direct-current (HVDC) system for carbon-neutral power transmission. The HCC accesses renewable power eliminating commutation failure through its hybrid operation modes of forced commutation and recovery-enhanced commutation, with the novelty of hybrid device connection topology. With the help of editors and reviewers, we have conducted a more in-depth study of the commutation principles and proposed the operational characteristics of the HCC-HVDC system. Finally, we build a 120 kV/360 MW HCC, which is used for the Lingbao super-HVDC project in Henan, China. The long-term engineering operation results prove it the state-of-the-art high-power converter.

Thanks for the attention of our paper. Looking forward to hearing from you.

Best regards,

Zhanqing Yu, Professor
Department of Electrical Engineering, Tsinghua University.

Rong Zeng, Professor
Department of Electrical Engineering, Tsinghua University.

Response to the Reviewer #1

We are grateful for the reviewer's constructive suggestions. We have responded diligently and meticulously, making extensive revisions addressing your concerns. If you have any further inquiries or require additional modifications, we would be happy to consider them. For ease of reference, the reviewer's comments are reproduced in ***black*** and our responses are given in ***blue***. The revised portions are marked in ***red*** in the new manuscript and the response letter.

Overall Comment: *The paper proposes a novel hybrid HVDC converter topology that integrates both IGCTs and thyristors. The methodology is thoroughly validated through experiments and simulation studies, and the evaluation has been carried out at a sufficiently practical scale. The paper is well-structured, and the potential impact of the proposed topology appears to be significant. My only major concern relates to the economic assessment of the proposed solution.*

Thank you for recognizing the authors' work and providing constructive comments. In response to your concerns about economic assessment, the authors have conducted thorough research and provided a comparison between the topology proposed in this manuscript and traditional solutions, as well as methods developed in previous studies. In accordance with the reviewer's suggestions, the comparison is presented in a large table.

Comment 1: *The proposed topology should be benchmarked against conventional alternatives through an economic-benefit analysis to clearly demonstrate its cost-effectiveness.*

Thank you very much for your suggestion. Based on this comment, the authors conducted a comparative economic analysis of existing ultra-HVDC transmission technologies, covering existing engineering projects and a literature search. The existing ultra-HVDC transmission technologies include the traditional LCC, VSC (including MMC), CLCC (Controllable Line Commutated Converter) [1], SLCC (Statcom and Line Commutated Converter) [2], and the HCC proposed in this article. The authors conducted a comparative analysis on the construction cost, operating losses, and footprint of converter stations.

In terms of the construction cost of converter stations, VSC uses a significant number of high-voltage and high-power IGBT devices. This eliminates the need for AC filter fields, but still has the highest total cost because of the large quantity of IGBT devices. LCC has the lowest converter construction cost and overall investment cost because of the small number and low unit price of thyristor devices. Among other technical routes, CLCCs and SLCCs have costs that lie between those of LCC and VSC

because of the increased number of auxiliary devices and the high cost of reactive power compensation equipment, respectively. The proposed HCC has a slightly higher cost than LCC because of the use of IGCTs, which cost slightly more than thyristors. Taking the ±800 kV ultra-HVDC project in China as an example, the total cost of a single LCC station is 5.5 billion yuan, with the unit cost basically controlled at 600–800 yuan/kW, while the total cost of a single VSC station is 7.5 billion yuan, giving a unit cost of 900–1300 yuan/kW. **Specifically, in terms of the cost of the valve, the costs of LCC, HCC, and VSC are 0.698, 0.838, and 2.176 billion yuan, respectively.**

In terms of operational losses, LCC has the lowest loss, with single station loss accounting for approximately 0.7%. VSC has the highest loss, with single station loss accounting for 1.2%. HCC has a slightly higher overall loss than LCC because of the higher loss in IGCTs than in thyristors. Compared with LCC, CLCC suffers ~25% higher losses, mainly through the on-state loss of the main branch IGBTs. The SVG part of SLCC has a higher operating loss, although the total loss of SLCC is slightly lower than that of VSC. **Specifically, in terms of valve losses alone, the losses of LCC, HCC, and VSC are 0.22%, 0.25%, and 0.7%, respectively.**

In terms of land area, the layout of LCC, CLCC, and HCC converter stations is similar, covering basically the same area. The presence of the AC filter field means that the land area reaches ~20 hectares. SLCC does not require this AC filter field, but adds SVG, occupying a similar area as LCC. Although VSC saves space in the AC filter field, the valve hall occupies a relatively large area. The ultra-high voltage flexible DC converter station currently occupies a total land area that is not less than that of LCC.

A comparison table is presented below, where it can be seen that HCC is **the most economical solution in topologies without commutation failures**.

TABLE I

COMPARISON OF DIFFERENT TECHNOLOGIES BASED ON ±800KV ULTRA-HVDC PROJECT

	LCC	**HCC**	CLCC	SLCC	VSC
Cost/10^8 CNY	53	**56**	58.5	57	70
Valve Cost/10^8 CNY	6.98	**8.38**	-	-	21.76
Loss/%	0.75	**0.79**	0.79	1.1	1.2
Valve Loss/%	0.22%	**0.25%**	-	-	0.70%
Area/hectares	20.17	**20.17**	20.17	20	26

Comment 2: *In particular, comparison with the most widely adopted hybrid HVDC configuration (LCC on the grid side and MMC on the offshore side) is essential. The advantages of the proposed approach, especially in terms of economic performance, should be explicitly*

discussed. I recommend large table to compare.

Thank you very much for your suggestion. In Table I, the authors have provided a quantitative economic benefit comparison of different topologies in ultra-HVDC applications. To demonstrate the advantages of the proposed HCC topology over traditional LCC and MMC systems, further comparisons are presented in Table II. From Table II, it is clear that **HCC retains the advantages of LCC's small capacitance, low loss, low cost, and high reliability**, while utilizing the fully controlled characteristics of IGCT to **solve the problem of commutation failure**, which is vital in next-generation HVDC systems.

TABLE II

COMPARISON OF TECHNICAL PARAMETERS AMONG LCC, HCC, AND MMC

Parameters	LCC on the grid side	**HCC on both sides**	MMC on the offshore side
Capacitor	μF	**μF**	mF
Commutation failure	With	**Without**	Without
Valve loss	0.22%	**0.26%**	1.00%
Station loss	0.75%	**0.79%**	1.20%
Reactive power compensation	Large	**Small**	Small
Harmonic	Low	**Low**	Medium
Valve power density	High	**High**	Medium
Valve Cost	1 p.u.	**1.2 p.u.**	3.12 p.u.
Control system complexity	Low	**Low**	High
Overall failure rate	Low	**Low**	High

[1] C. Gao et al., "Novel Controllable-Line-Commutated Converter for Eliminating Commutation Failures of LCC-HVDC System," in IEEE Transactions on Power Delivery, vol. 38, no. 1, pp. 255-267, Feb. 2023.

[2] R. Xu et al., "Analysis and control method of SLCC-HVDC system oriented to retrofit AC transmission lines," 2024 3rd International Conference on Power Systems and Electrical Technology (PSET), Tokyo, Japan, 2024, pp. 268-272.

In direct response to this comment, we have added analysis in the **Discussion** section. This revised text provides a systematic comparison based on established project metrics.

" **The economic argument for HCC is compelling, particularly when viewed through the lens of lifecycle cost and its role in enabling carbon neutrality. A comparative analysis is summarized in Table 1. For long-distance, large-scale renewable energy transmission projects, such as delivering power from remote**

"sand-gobi-desert" renewable bases in China or similar settings worldwide, the cost per megawatt-kilometer is a decisive factor. The economic profile of the HCC, which approaches that of LCCs while offering VSC-like stability, significantly reduces the levelized cost of electricity for clean energy. By providing a reliable, high-capacity corridor without the risk of commutation-failure-induced outages, the HCC enhances grid utilization and reduces the need for redundant backup infrastructure. This improved efficiency and reliability directly translate into lower system-wide costs and accelerated deployment of renewable energy, thereby delivering substantial carbon mitigation benefits over the project's lifetime. "

Table. 1 Comparative Analysis of HVDC Converter Technologies

Aspect	LCC	VSC(MMC)	LCC-VSC Hybrid	HCC (This work)
Capital Cost	Low	High	Very High	Low (close to LCC)
Operational Losses	Low	High	Medium-High	Low (inherited from LCC)
Commutation Failure Risk	High	None	Low	None (Active immunity)
Reactive Power Support	Consumer (requires external compensation)	Full, independent control	Provided by VSC part	Controllable support
System Complexity	Low	Medium	Very high	Low
Key Enabler for Renewables	Limited by stability concerns	Excellent but costly	Effective but expensive	High-capacity, stable & cost-effective

Thanks for the valuable comments and for exchanging academic and engineering views with the reviewer. The manuscript is improved a lot through the academic discussions. The authors analyzed and discussed seriously all the comments and questions raised by the reviewer. The descriptions, explanations, and comprehensive analysis are deepened and added to improve the quality of the study, which we hope, has addressed the concerns of the reviewer.

Response to the Reviewer #2

We are grateful for the reviewer's constructive suggestions. We have responded diligently and meticulously, making extensive revisions addressing your concerns. If you have any further inquiries or require additional modifications, we would be happy to consider them. For ease of reference, the reviewer's comments are reproduced in ***black*** and our responses are given in ***blue***. The revised portions are marked in ***red*** in the new manuscript and the response letter.

Overall Comment: *This paper proposes a High-Power hybrid-commutated converter (HCC) HVDC system to address the commutation failure issue in conventional LCC-HVDC systems through the hybrid operation modes of forced commutation and recovery-enhanced commutation. Both simulation and test results have been shown in this paper and this HCC technology has been applied in a real HVDC project in China. All of these demonstrate that it is practical research. However, this work should be compared with existing works to highlight its contribution and novelty.*

We sincerely appreciate the reviewer's positive assessment of our manuscript. In response to your comments, the authors **have conducted an additional literature search to better demonstrate the novelty of the proposed HCC topology**. Furthermore, **all technical concerns raised have been thoroughly addressed** with corresponding revisions and supplementary explanations in the revised manuscript.

Comment 1: *Abstract (P1, Line 5): the statement 'however, they still face the risk of commutation failure, threatening the safety of the power grid' is true in the LCC-HVDC system and this issue has been widely discussed in the literature. However, it does not apply to VSC-HVDC systems. The author should clarify this to avoid unnecessary confusion.*

Thank you very much for your comment. LCC has commutation failure issues, while VSC has limitations in terms of cost and reliability. To avoid unnecessary confusion, the authors have changed this statement in the **Abstract** to "**However, the former suffer from a risk of commutation failure, threatening the safety of the power grid, while the latter entail higher carbon emissions, lower robustness, and higher power loss.**"

Comment 2: *Section following the abstract (P1) authors introduce the HVDC development in China, the impact of commutation failure in the LCC system, limitation of MMC-HVDC. However, there is no comparison with existing works? Has any research been done in this area? If so, please review existing methods and highlight the*

contribution/novelty of this paper.

Apart from second-generation HVDC systems, there has been extensive research on the commutation failures encountered by first-generation HVDC systems. Existing studies include capacitor-based topologies, SLCC, and CLCC. Following the reviewer's suggestion, the authors have added a technical comparison section in the manuscript: "**Numerous studies have explored the mitigation of commutation failures and enhanced power system stability. Capacitor-based topologies, such as Capacitor Commutated Converters (CCCs) [30], Controlled Series Capacitor Converters (CSCCs) [31], and Evolutional Line-Commutated Converters (ELCCs) [32], use series capacitors to support the voltage under fault conditions, thereby reducing the risk of commutation failures. However, their effectiveness in resisting commutation failure is limited, and the additional capacitors introduce new harmonic issues. Statcom and Line Commutated Converters (SLCCs) employ Static Var Generators (SVGs) connected in parallel on the AC side of the converter to support the grid by controlling the reactive power output of the SVG [33]. Controllable Line Commutated Converters (CLCCs) use additional auxiliary bridge arms to actively interrupt the current, which can resolve commutation failures, but the incorporation of a large number of IGBT devices results in additional costs [34].**"

Comment 3: *Result A (P3, Line 23 to 26): authors propose that the HCC combines both MMCs and LCCs, thus leveraging the advantages of MMCs and LCCs. Please specify what advantages of MMCs and LCCs are being used. In addition, as it uses a hybrid structure, are there any limitations or trade-offs compared to the pure LCC or MMC system? This clarification could help readers understand the proposed technology.*

LCC offers advantages such as large capacity, low power losses, and high reliability, but it suffers from commutation failure issues. MMC, on the other hand, eliminates the risk of commutation failure, yet it is characterized by high carbon emissions, low robustness, and high losses. HCC employs a hybrid configuration of fully-controlled and semi-controlled devices, inheriting the advantages of LCC such as large capacity, low losses, and high reliability, while leveraging fully-controlled devices to achieve immunity to commutation failure during AC faults. The authors have added clarification: '...which combines the principles of MMCs and LCCs. **During stable system operation, HCC adopts the semi-controlled commutation principle of LCC, thereby inheriting LCC's advantages of large capacity, low losses, and high**

reliability. Under fault conditions, HCC uses the fully controlled commutation principle of MMC to achieve immunity to commutation failures. The proposed HCC...'.

The distinction between HCC and LCC lies solely in the device-level composition. Specifically, IGCTs incorporate a gate drive controller based on conventional thyristors, thereby enabling active turn-off capability. This increased control freedom inevitably leads to reduced reliability. Consequently, compared with pure LCC, HCC achieves immunity to commutation failures at the cost of a marginal reduction in reliability. In contrast to MMC, which employs modulation waves to control frequent bridge arm switching, HCC preserves the advantages of low losses and high reliability by only using active turn-off under fault conditions to mitigate commutation failures. As a result, HCC does not possess the decoupled active and reactive power control capability or transient voltage support capability that MMC offers. However, these limitations and trade-offs do not affect the application of HCC in the context discussed in this manuscript, and therefore the authors have not included any additional elaboration. The authors have added the following clarification in the **Discussion** section:

"**However, it is essential to acknowledge the current limitations of the HCC technology. The primary constraint is reduced flexibility compared with a full MMC. Furthermore, the HCC operates as a grid-following converter with voltage-support capability, and the grid-forming capability of it is still under research. Consequently, its optimal current application is in high-capacity, point-to-point or multi-terminal transmission corridors, solving the commutation failure problem rather than meeting the high flexibility demand. Future work will focus on exploring partial modularization within the efficient HCC architecture and refining the associated control strategies.**"

Comment 4: *Result B (P4, Line 17-18: the di/dt of current on each bridge was used to trigger the forced commutation principle. Theoretically, it should use the point where di/dt=0. However, considering the disturbances in real projects like noises, a threshold of 0 might lead to the maloperation of the forced commutation as it is too sensitive. Please clarify, 1) how to set the threshold of di/dt in real implementation? What factors should be considered during the threshold selection?*

We sincerely thank the reviewer for raising this important practical consideration regarding the di/dt threshold setting. The comment correctly identifies the challenge of implementing the forced commutation principle under realistic conditions with measurement disturbances.

As clearly stated in the manuscript, during the commutation failure process, "i_{an}

and i_{bn} both have a period for which di/dt = 0. This special characteristic of the current change rate creates an opportunity for the intervention of the forced commutation principle". However, the current waveform oscillations in practical projects make the criterion of di/dt == 0 unattainable. In our practical implementation, the di/dt threshold was set at **0.5 A/μs**. This value was determined through extensive electromagnetic transient simulations and validated by field testing in the Lingbao HVDC project. It represents the optimal balance between sensitivity to genuine commutation failures and immunity against measurement noise and current waveform oscillations.

The selection of this threshold was primarily influenced by three critical factors: **current waveform quality**, **sensor performance limitations**, and **control complexity**. The inherent oscillations and harmonics in the current waveform during both normal and fault conditions necessitate a nonzero threshold to avoid false triggers. The measurement accuracy and resolution of the optical current sensors (specified as 1 A at 250 kHz in our setup) directly impact the smallest detectable and reliable di/dt value. A higher threshold simplifies the control logic and enhances robustness, but an excessively high value could delay or miss the detection of a real commutation failure. The chosen 0.5 A/μs provides a sufficient safety margin against noise while ensuring rapid and reliable detection. Furthermore, to enhance robustness against transient disturbances, our implementation incorporates a **20-sample confirmation logic**. The forced commutation is triggered only when the di/dt value exceeds the 0.5 A/μs threshold for 20 consecutive sampling points (equivalent to a duration of 80 μs at our 250 kHz sampling rate). This filtering mechanism effectively discriminates between noise spikes and a genuine commutation failure event.

The authors have made the following additions to the manuscript. "Current sensors, **with a resolution of 1 A and a sampling rate of 250 kHz**, can thus be installed on each bridge of the HCC for di/dt detection. **Considering the inherent oscillations and harmonics in the current waveform, our implementation incorporates a 20-sample confirmation logic. The forced commutation is triggered when d*i*/d*t* exceeds the 0.5 A/μs threshold for 20 consecutive sampling points**, actively breaking the fault current at time t_4. **Thus, the time period from t_3 to t_4 depends on the detection speed of the current sensors, which is 80 μs**."

Comment 5: *More context should be provided to explain the results in Fig 2.C. For example, why is a larger discrepancy between the test and simulation results observed on the blue curve?*

We thank the reviewer for this insightful comment regarding the discrepancy observed in Fig. 2c. The point is valid and allows us to provide a more detailed explanation of the modeling strategy and its inherent trade-offs, which we have now

clarified in the revised manuscript.

The observed discrepancy, particularly evident in the later phase of the blue curve (representing the voltage redistribution process), stems from a deliberate and justified trade-off in our modeling approach. To make system-level simulation of the HCC feasible within a reasonable computation time, we employed a **physics-based compact model** that prioritizes simulation speed over full physical detail. This model simplifies the internal semiconductor physics by segmenting an IGCT into six blocks for modular modeling.

Consequently, while this compact model achieves high fidelity in simulating the **critical active turn-off transient**, which is the primary focus of our analysis and is essential for validating the HCC's core functionality, it inherently sacrifices accuracy in simulating secondary processes such as the subsequent **voltage redistribution**. The larger discrepancy in the blue curve is a direct result of this prioritization; the model's simplified representation does not capture the full complexity of the internal charge dynamics and parasitic effects that govern the long-tail voltage behavior with the same precision as the initial, more critical turn-off event.

The following clarification has been added to the manuscript: "... as shown in Fig. 2c. **By adopting a physics-based compact model for faster computation, the accuracy of voltage redistribution in subsequent processes was sacrificed to preserve the fidelity of the more critical active turn-OFF transient simulation.** To further investigate... "

Comment 6: *Discussion (P7, Line 75-83): this part states that the HCC-HVDC system is effective in long-distance and large-scale renewable energy transmission. Why in this paper a 120kV/360 MW HVDC link used as an example? The voltage and power in this link are not too high compared to the UHVDC (≥800 kV), as mentioned by the author.*

We thank the reviewer for this pertinent observation regarding the system parameters. The selection of the 120 kV/360 MW case study is based on its status as the **first practical implementation of HCC technology** in a real-world HVDC project (the Lingbao HVDC link). While we fully acknowledge that its voltage and power ratings are below those of UHVDC systems, this specific project serves as the foundational (and currently only) available validation platform for the proposed topology.

Using this real operational system as an example allows us to present experimentally validated results rather than purely simulated projections, which significantly strengthens the technical credibility of our findings. The core operational principles, control strategies, and performance advantages of the HCC—particularly its

immunity to commutation failures and flexible reactive power control—are successfully demonstrated at this scale. These characteristics are inherently scalable and form the critical foundation for extending the HCC topology to higher-voltage, higher-power applications, including future UHVDC systems.

The authors have included photographs of the actual valve tower from the project and the field conditions during the short-circuit test of the HCC converter, as shown in Fig. A and Fig. B.

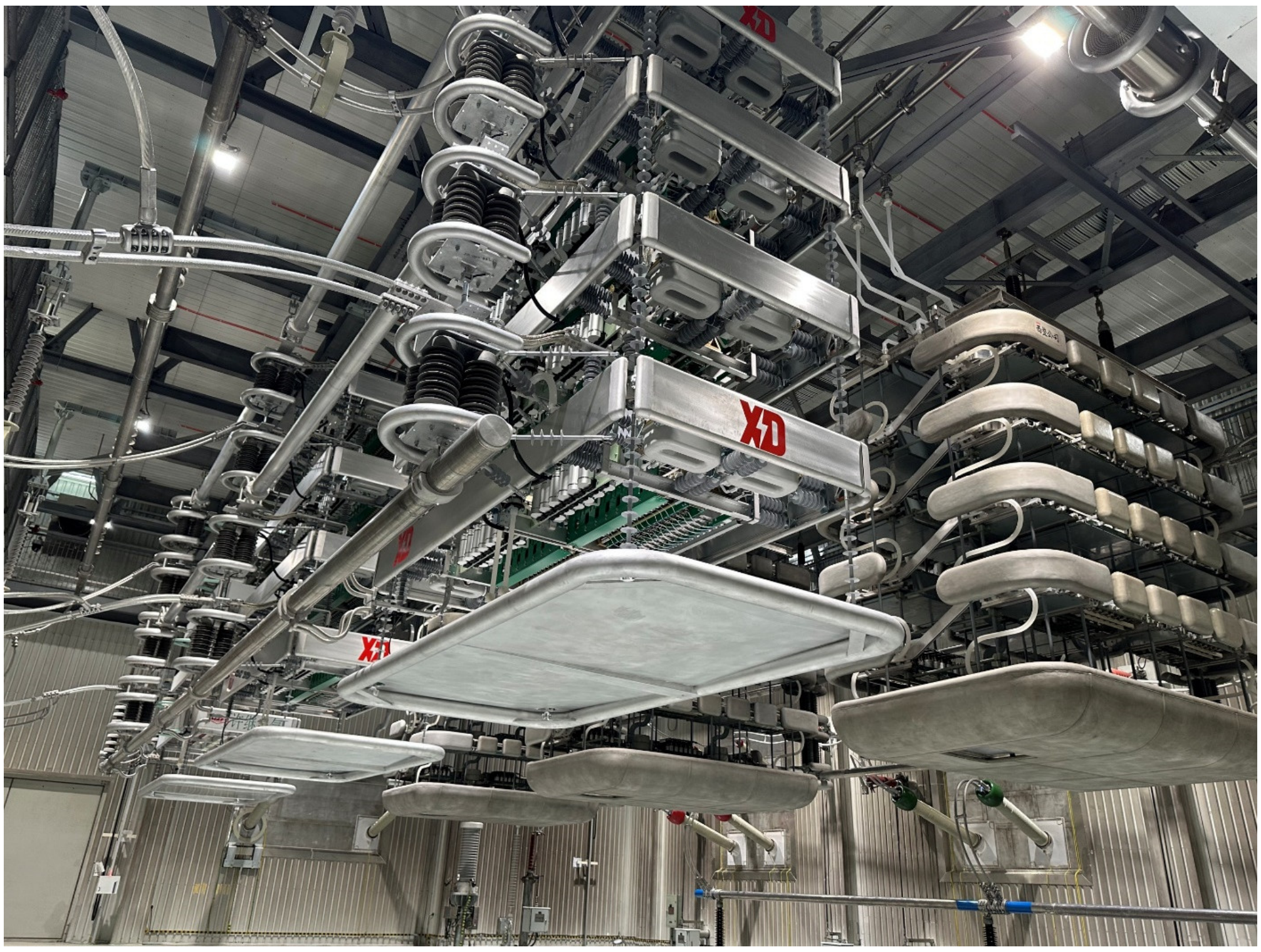

Fig. A The photograph of the actual valve tower in the Lingbao project in Henan, China

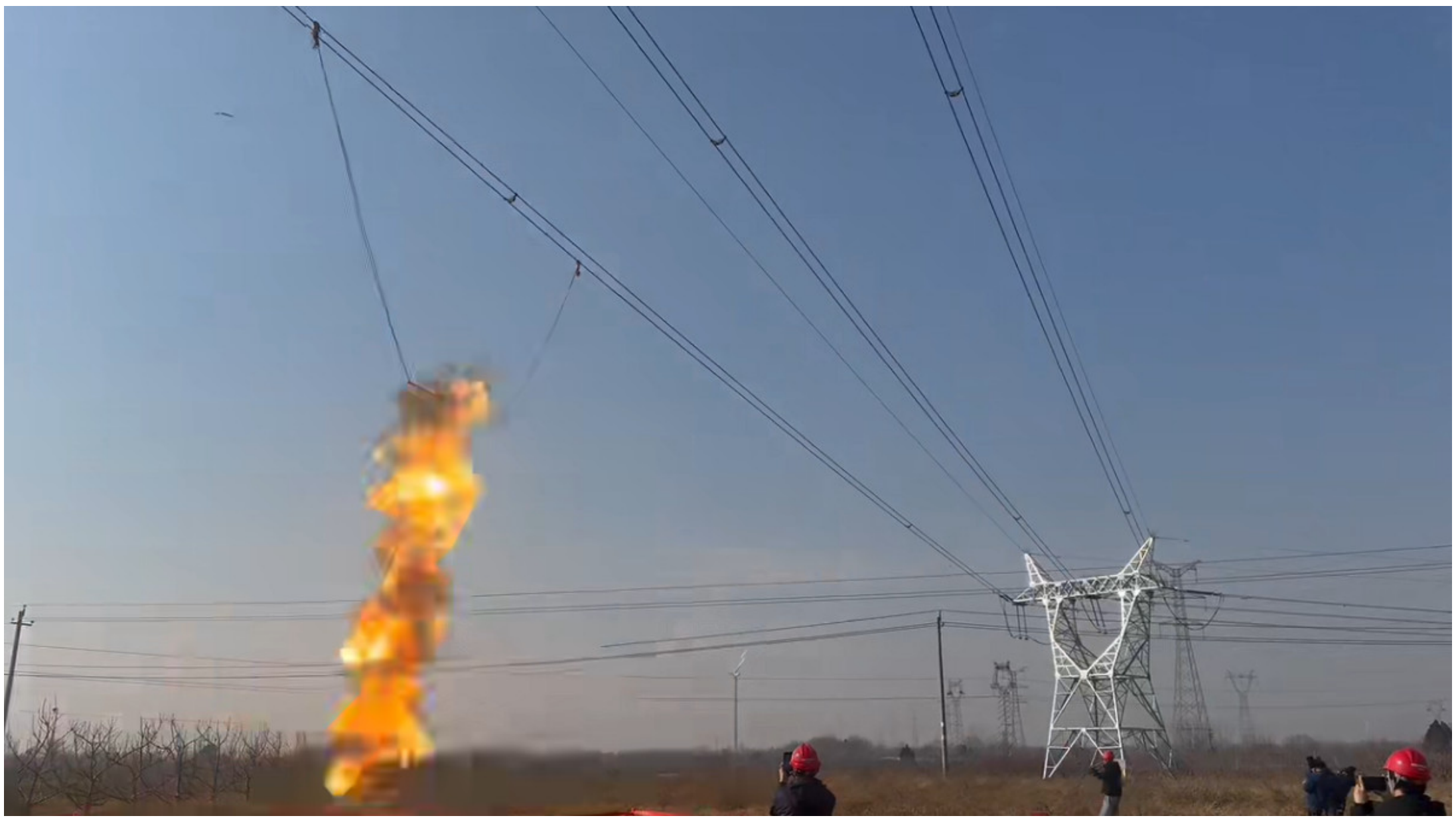

Fig. B The photograph of the field conditions during the short-circuit test of the HCC system in Henan, China

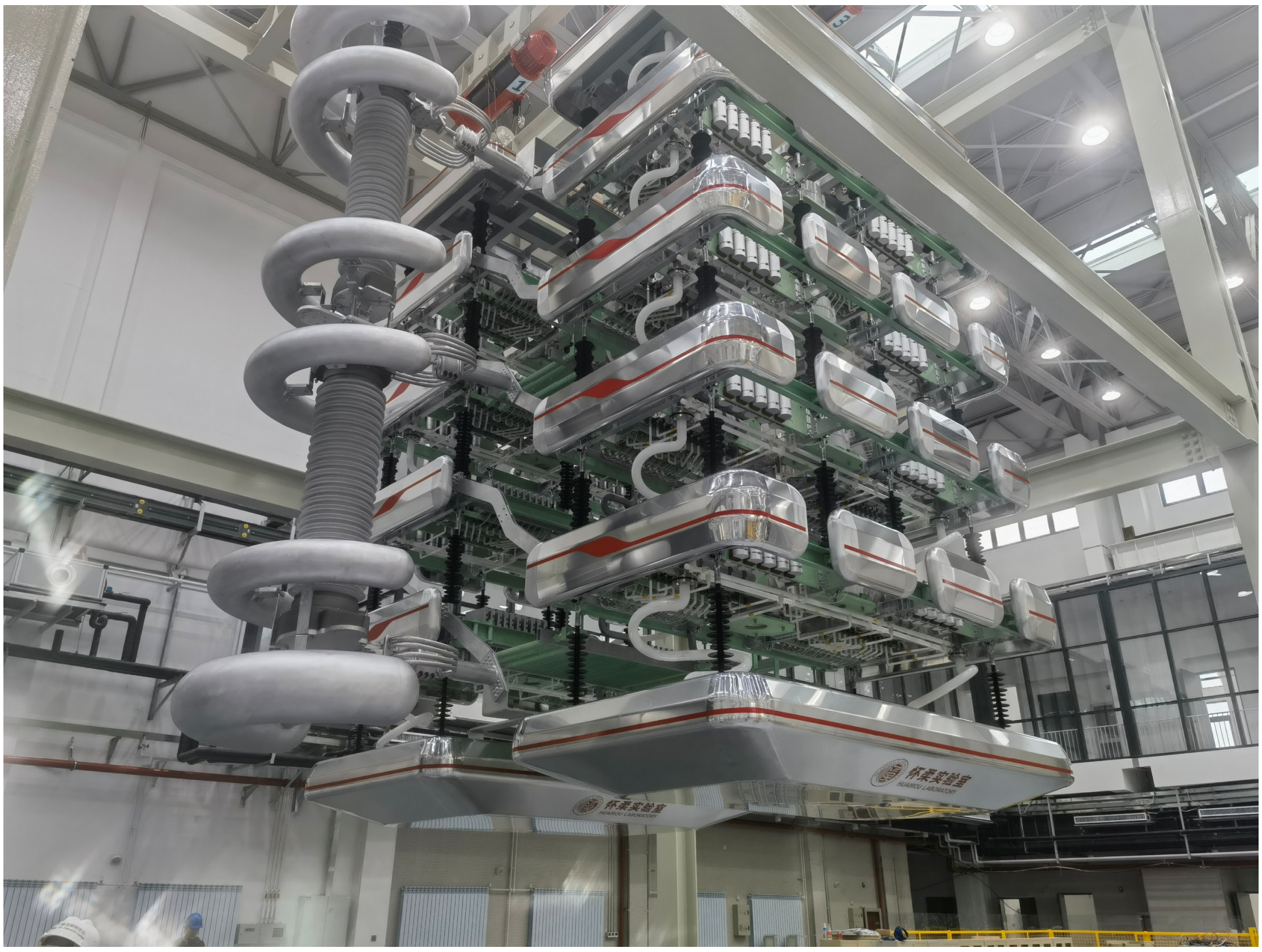

Fig. C The UHVDC HCC valve tower

Following the successful implementation of the Lingbao super-HVDC HCC project, the authors are now advancing the development of a ultra-HVDC HCC system.

To date, the electrical parameter design for the UHVDC HCC has been completed, and a prototype valve tower has been manufactured, as shown in Fig. C. Concurrently, the corresponding single-valve operational tests have been fully concluded. The HCC will be used in Mengxi ultra-HVDC project in Inner Mongolia, China in 2027.

Comment 7: *The arrows in Figure 5 should be plotted using professional plotting tools, rather than the hand-drawn.*

We thank the reviewer for this valuable feedback regarding the presentation quality of Fig. 5. We agree that professional visualization is crucial for clarity. In response, we have re-plotted the figure.

Thanks for the valuable comments and for exchanging academic and engineering views with the reviewer. The manuscript is improved a lot through the academic discussions. The authors analyzed and discussed seriously all the comments and questions raised by the reviewer. The descriptions, explanations, and comprehensive analysis are deepened and added to improve the quality of the study, which we hope, has addressed the concerns of the reviewer.

Response to the Reviewer #3

We would like to thank the reviewer's constructive suggestions. We have responded diligently and meticulously, making extensive revisions addressing your concerns. If you have any further inquiries or require additional modifications, we would be happy to revise them. For ease of referencing, the reviewers' comments are reproduced in ***black*** and our responses are given in ***blue***. The revised portions are marked in ***red*** in the new manuscript and the response letter.

Overall Comment: *The topic of this paper, dealing with relatively new HVDC converter technology, is both relevant and timely, given the growing importance of HVDC systems. The authors have described the technology, and the presented results in the manuscript can be demonstrated by attached materials. Nevertheless, this reviewer identifies several critical points that should be addressed to further strengthen the contribution.*

We sincerely thank the reviewer for this positive assessment of our work's relevance and for the valuable feedback. The comments have been highly insightful in helping us strengthen the manuscript. Specifically, a more detailed comparative analysis with existing hybrid topologies has been added in the **Introduction**, the innovation of our study has been re-emphasized, and operational characteristics of the HCC system have been added to the manuscript. We have carefully addressed all the points raised in the revised version. The authors believe these revisions significantly enhance the technical depth and clarity of our contribution.

Comment 1: *Scope of literature review*

The current manuscript appears to focus mainly on the status of HVDC deployment in a specific country. While it is understandable that most HVDC applications have been concentrated there, a broader overview covering worldwide developments would be highly beneficial for the readers. For instance, European projects often provide publicly available information that could help balance the review. Since many readers and reviewers may not have access to the same depth of information from that specific country as much as authors can do, a more global perspective would enhance the completeness of the manuscript.

We sincerely thank the reviewer for this insightful comment regarding the need for a more global perspective on HVDC deployment. We fully agree that broadening the scope would significantly enhance the manuscript's value for the international

readership. In response to this valuable suggestion, we conducted research on HVDC projects and planning worldwide, providing a concise yet comprehensive overview of major HVDC developments beyond China, including Europe, the United States, and Brazil.

Europe's HVDC development is strategically driven by its carbon neutrality goals and energy security strategy, with a clear focus on integrating distributed renewable energy—particularly North Sea offshore wind and southern European solar power—through cross-border grid interconnections. While early projects used LCC technology, VSC systems, especially MMC, have become the mainstream solution for new projects because of their superior controllability, absence of commutation failure risk, and flexibility in forming multi-terminal systems. Looking forward, Europe is actively advancing toward a pan-European HVDC "super grid" overlay, with strong emphasis on multi-terminal systems and offshore DC grids.

In the **United States**, HVDC deployment is primarily motivated by the need to address internal transmission bottlenecks, enhance grid resilience, and integrate remote renewable generation. Current applications include back-to-back interties for connecting asynchronous grids and long-distance point-to-point transmission, with emerging use-cases in powering high-density AI data centers to improve energy efficiency. Future development is strongly oriented toward offshore wind transmission, as outlined in the Atlantic Offshore Wind Transmission Plan, which envisions regional coordination and HVDC links.

Brazil's HVDC expansion is fundamentally shaped by the need to transmit large-scale hydropower and renewable resources from the remote north and northwest to load centers in the southeast over ultra-long distances. The country has successfully implemented ±800 kV UHVDC technology in major projects and continues to expand its HVDC network to integrate growing solar and wind capacity, as detailed in its 2025 Power Transmission Plan and 2034 Ten-Year Energy Expansion Plan. A critical future challenge involves addressing the projected need for 35 GW of peak shaving capacity by 2034, creating significant opportunities for energy storage deployment. Brazil is also actively leveraging international cooperation, notably through joint innovation platforms with Chinese partners, to introduce advanced technologies in renewable integration and smart grid solutions.

To give a broader overview of worldwide developments in HVDC systems, the authors have added the following content in the Introduction: "**Globally, the deployment of HVDC systems is being strategically advanced to address diverse regional energy challenges. Europe is focusing on cross-border interconnection and renewable integration through an emerging "super grid" based on VSC-**

HVDC technology, particularly from North Sea offshore wind power and southern European solar power. The US is prioritizing the resolution of internal transmission bottlenecks and enhanced grid resilience. At present, the US employs HVDC in applications ranging from back-to-back interties to long-distance transmission, supported by initiatives such as the Atlantic Offshore Wind Transmission Plan. Brazil has employed HVDC systems, including ±800 kV ultra-HVDC projects, to transmit large-scale hydropower and growing renewable resources from remote northern regions to southeastern load centers, guided by its national energy expansion plans and international collaborations for tackling future challenges such as peak shaving and smart grid integration."

The comprehensive analysis of HVDC development pathways in Europe, the United States, and Brazil demonstrates that HCC technology holds significant potential as a key grid technology for the future: it provides an optimal balance between cost-effectiveness and resilience for Europe's super grid vision, offers a simplified solution to the complex DC protection challenges faced by the United States, and presents an optimal solution balancing economy and stability for Brazil's long-distance transmission needs, highlighting its broad applicability and unique value across different regional contexts.

Comment 2: *Clarification of novelty*

The novelty of the paper is not sufficiently clear. Is the manuscript intended as a review paper? If so, it should more closely follow the structure and depth of a review article, including a comprehensive overview of the current state of the field. Alternatively, if the novelty lies in introducing a new generation of HVDC converters (such as HCC), then this needs to be clearly articulated. As it stands, the paper leaves some ambiguity, particularly since there already exist numerous publications on the same topic, including from the authors.

We sincerely thank the reviewer for raising this critical point regarding the novelty and positioning of our manuscript. The comment provides us with an excellent opportunity to clarify our core contribution.

The manuscript indeed falls into the second category outlined by the reviewer: it introduces and validates a **new generation of HVDC converter, the HCC**, as a complete system-level innovation. While our previous publications, rightly noted by the reviewer, focused on specific scientific aspects such as the *rapid commutation principle*, *low reactive power operation*, and *voltage balancing methods*, this manuscript represents a significant step forward.

The primary novelty of this work lies in the **integrated presentation,**

development, and full-scale experimental validation of the complete HCC system. We have moved beyond analyzing individual technical points to demonstrating how these innovations combine to form a viable and superior converter topology. This includes the holistic topology design, the engineering implementation of the converter prototype, and crucially, comprehensive test results that validate its performance as a unified system under various operational and fault conditions. Therefore, this paper is not intended as a review, but as a foundational article that establishes the HCC as a new, fully realized member of the HVDC converter family. We have revised the introduction and discussions to articulate this system-level novelty and contribution more unequivocally.

Introduction: "... their respective disadvantages. **The primary novelty of this work lies in the integrated presentation, development, and full-scale experimental validation of the complete HCC system. Experimental results from** a 120 kV/360 MW HCC..."

Discussion: "... **The principal innovation of this work lies in the holonic development and validation of the complete HCC system, moving beyond theoretical concepts or isolated component studies. This encompasses the novel topology design, the development of key operational principles, the engineering realization of a full-scale prototype (e.g., the 120 kV/360 MW system), and its comprehensive experimental validation. This system-level proof is crucial for transitioning from academic research to industrial application...**."

Comment 3: *Literature review on HCC and related technologies*

Closely related to the previous point, the manuscript currently lacks a thorough literature review of existing work on HCC. Given that many relevant publications are available, including from the authors themselves, it is important to situate the present work within that context. Furthermore, as the authors emphasize HCC's high-capacity potential, it would be valuable to review previous efforts addressing comparable challenges, such as hybrid LCC–VSC HVDC systems and bipole HVDC configurations.

The authors attach great importance to the reviewer's comments on the LCC-VSC hybrid structure and have conducted corresponding research.

The LCC-VSC series topology, shown in Fig. D, is fundamentally characterized by the summation of voltages on the DC side. This configuration connects a cost-effective, low-loss LCC, responsible for handling the primary high voltage and power, in series with a VSC that bears a portion of the voltage while providing crucial control capabilities. This achieves a balance between UHVDC power transmission and economic viability. Its most prominent feature is the use of the VSC as a "voltage

supporter", which **actively maintains the DC voltage during faults in the receiving-end AC grid, thereby reducing the commutation failure rate of the LCC**. Additionally, the VSC provides enhanced functionalities such as dynamic reactive power support and oscillation damping for the system. **However, a significant drawback of this topology lies in its highly complex coordination control and protection configuration.** A fault in any converter station affects the entire system, and the VSC section must withstand the full DC fault current, **imposing stringent requirements on the overcurrent capability of its semiconductor devices.**

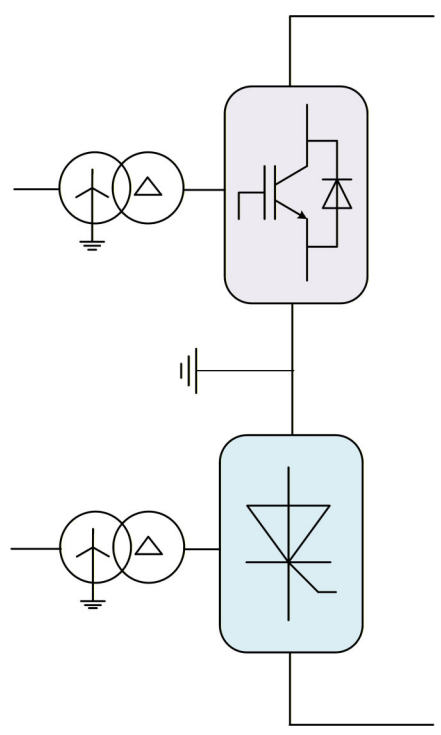

Fig. D The LCC-VSC series topology

The LCC-VSC parallel topology, shown in Fig. E, is characterized by the convergence of currents and the summation of power on the common DC bus. This structure connects both LCC and VSC converters to the same DC bus, enabling increased transmission capacity and complementary functionalities. Its greatest advantage lies in its **exceptional flexibility and redundancy**. The topology not only facilitates the straightforward formation of multi-terminal DC systems for flexible power dispatch, but also enables "N−1" operation, allowing the system to continue operating at a reduced capacity if a single converter station fails, thereby significantly enhancing power supply reliability. **The primary disadvantages of this topology are concentrated in DC voltage coordination control and fault management.** It requires a highly coordinated control strategy to maintain voltage stability. Furthermore, during a DC-side short-circuit fault, the LCC continues to feed a substantial fault current, making fault clearance exceptionally difficult and typically **necessitating the use of costly high-speed DC circuit breakers.**

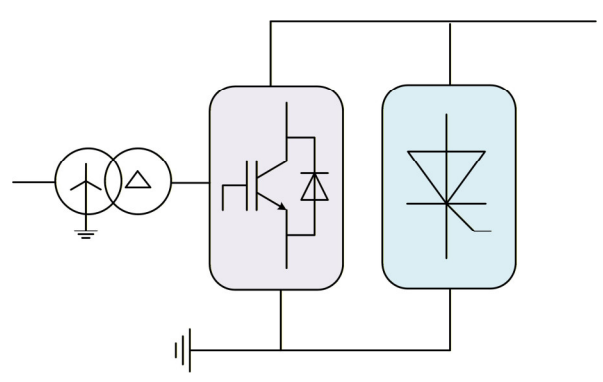

Fig. E The LCC-VSC series topology

Compared with LCC/VSC hybrid topologies, HCC achieves complete immunity

to commutation failures by replacing a portion of its components, resulting in lower costs and eliminating the need for complex control and protection configurations. Furthermore, because IGCT devices possess the same short-circuit current capacity as thyristors, HCC does not require DC circuit breakers.

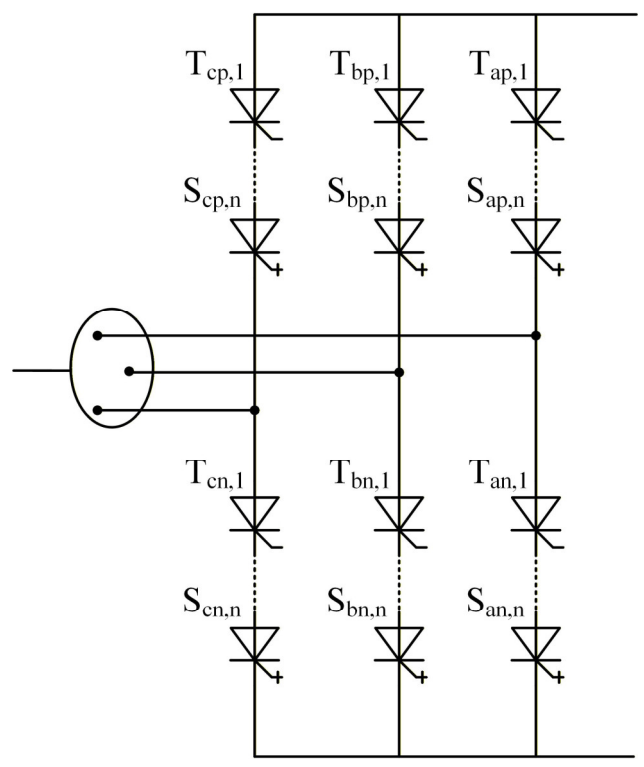

Fig. F The proposed HCC topology

We have revised the **Introduction** to include this comparison between HCC and LCC-VSC hybrid topologies. In the **Discussion**, the authors also provide an economic comparison between LCC, VSC, LCC-VSC hybrid, and HCC topologies.

"The LCC-VSC hybrid topology can partially mitigate commutation failure issues, but its system-level architecture is inherently complex and costly. Specifically, the LCC-VSC hybrid solution requires two completely independent converter systems, with their associated control systems. This leads to challenges in terms of coordinated control, a larger physical footprint, and the necessity of DC circuit breakers for effective fault management."

Comment 4: *Operational aspects beyond device-level characterization*

The current manuscript mainly discusses HCC from a component-level characteristics, but provides limited insight into its operational behavior. This aspect is really important to be attractive for the application to replace with currently available one such as LCC and VSC types. Therefore, fair comparison with LCC and VSC in terms of power system operational aspect is also should be highlighted otherwise, it is not attractive as authors insisted.

The reviewer suggests that including basic operational characteristics would strengthen the work. For example, an illustration of the basic control structures, dynamic responses to power reference changes. In addition, comparisons with LCC and VSC technologies—highlighting controllability, reactive power requirements, and operational flexibility—would better demonstrate HCC's advantages. E.g., a discussion of reactive power consumption would be highly informative for the comparison with

LCC. In short, reactive power characteristics compared to LCC & superior controllability for the active & reactive power compared to VSC.

We sincerely thank the reviewer for this critical and insightful comment regarding the analysis of operational behavior. We fully agree that demonstrating system-level performance is essential in highlighting the practical value and competitiveness of the proposed HCC technology against established LCC and VSC solutions.

In direct response to this valuable suggestion, we have undertaken a major revision to shift the focus from component-level to system-level characterization. Specifically, a **new dedicated section titled "Operational Characteristics of HCC System"** has been incorporated into the revised manuscript. This section comprehensively addresses the reviewer's concerns through the following key additions:

1. Illustration of Control Architecture: A detailed system control block diagram is now provided, elucidating the coordinated control strategy that manages the active turn-off converter. This framework forms the basis for its unique operational capabilities.

2. Analysis of Core Operational Advantages: The section provides an in-depth analysis of two fundamental operational strengths:

Commutation Failure Immunity: Building upon the control framework, we elaborate on the operational sequence of the "recovery-enhanced" and "forced" commutation modes. Dynamic response analyses under various AC fault conditions are presented to quantitatively demonstrate HCC's complete immunity to commutation failures, which is a decisive advantage over traditional LCC.

Reactive Power Capability: A dedicated comparative analysis highlights HCC's superior reactive power characteristics. Unlike an LCC, which is a net reactive power consumer and requires large external compensators, the HCC provides controllable reactive power support. The manuscript now includes quantitative results (e.g., its capacity to provide up to 0.5 p.u. of support during voltage dips), demonstrating the role of HCC in enhancing grid voltage stability, a function for which LCC is fundamentally limited.

We believe these substantial additions significantly strengthen the manuscript by providing the necessary system-level insight and comparative evidence. They clearly articulate why HCC is not merely a theoretical component improvement but a compelling and attractive alternative for practical HVDC applications, effectively addressing the core concern raised in this comment.

"**The commutation failure mitigation strategy employed in HCC is non-predictive. Consequently, discrimination between its two distinct operational modes, "forced commutation" and "recovery-enhanced commutation", relies on**

the real-time detection of temporal and current parameters, as illustrated in Fig. 4a. Upon the initiation of commutation, the control system opens a commutation window with a fixed duration, T0. If the current derivative (di/dt) remains negative throughout this window and the current decays to zero by the end of the window, the process is identified as recovery-enhanced commutation. Conversely, the system interprets a positive di/dt within the window or a nonzero current persisting at the window's terminus as an impending commutation failure. In this scenario, it issues a turn-OFF command and simultaneously checks the status of the turn-OFF enable signal. If this signal is active (logic '1'), the current is successfully interrupted, executing forced commutation. If the enable signal is inactive (logic '0'), commutation failure occurs.

The combined commutation strategy of recovery-enhanced and forced commutation provides effective immunity against commutation failures across all AC system fault conditions. A representative commutation failure mitigation process during a single-phase-to-ground fault is depicted in Fig. 4b. The data demonstrate that the LCC, lacking an inherent mitigation mechanism, suffers from successive commutation failures, where the current fails to transfer to bridge arms 1 and 5 (Va1 and Va5). In contrast, the HCC achieves orderly current commutation among three bridge arms via active turn-OFF. Figure 4c, 4d, and 4e present the maximum required turn-OFF current for successful mitigation during single-phase-, two-phase-, and three-phase-to-ground faults, respectively, under varying fault depths and initiation times. Analysis reveals that, in general, the required turn-OFF current increases with the number of faulted phases. Notably, the highest single demand for turn-OFF current, 5.15 kA, occurs under the two-phase-to-ground fault scenario. This indicates that an IGCT with a turn-OFF capability exceeding 5.15 kA enables the HCC to prevent commutation failures under all types of AC system faults.

Beyond immunity from commutation failures, the HCC exhibits a distinct advantage over the LCC in providing reactive power support to the grid during voltage sags. Figure 4f compares the performance of an LCC and an HCC under a 0.5 p.u. AC voltage dip. The HCC, endowed with robust reactive power support, maintains relatively stable voltage and current profiles, in stark contrast to the LCC. Figure 4g delineates the operational range of the HCC's reactive power support. By adjusting the extinction angle across different DC voltage levels, the HCC can flexibly switch between absorbing and supplying reactive power. A pivotal capability is demonstrated at 1 p.u. voltage with an extinction angle of −30°, where the HCC can supply reactive power support at up to 45.4% of its nominal capacity. This performance marks a significant breakthrough for maintaining

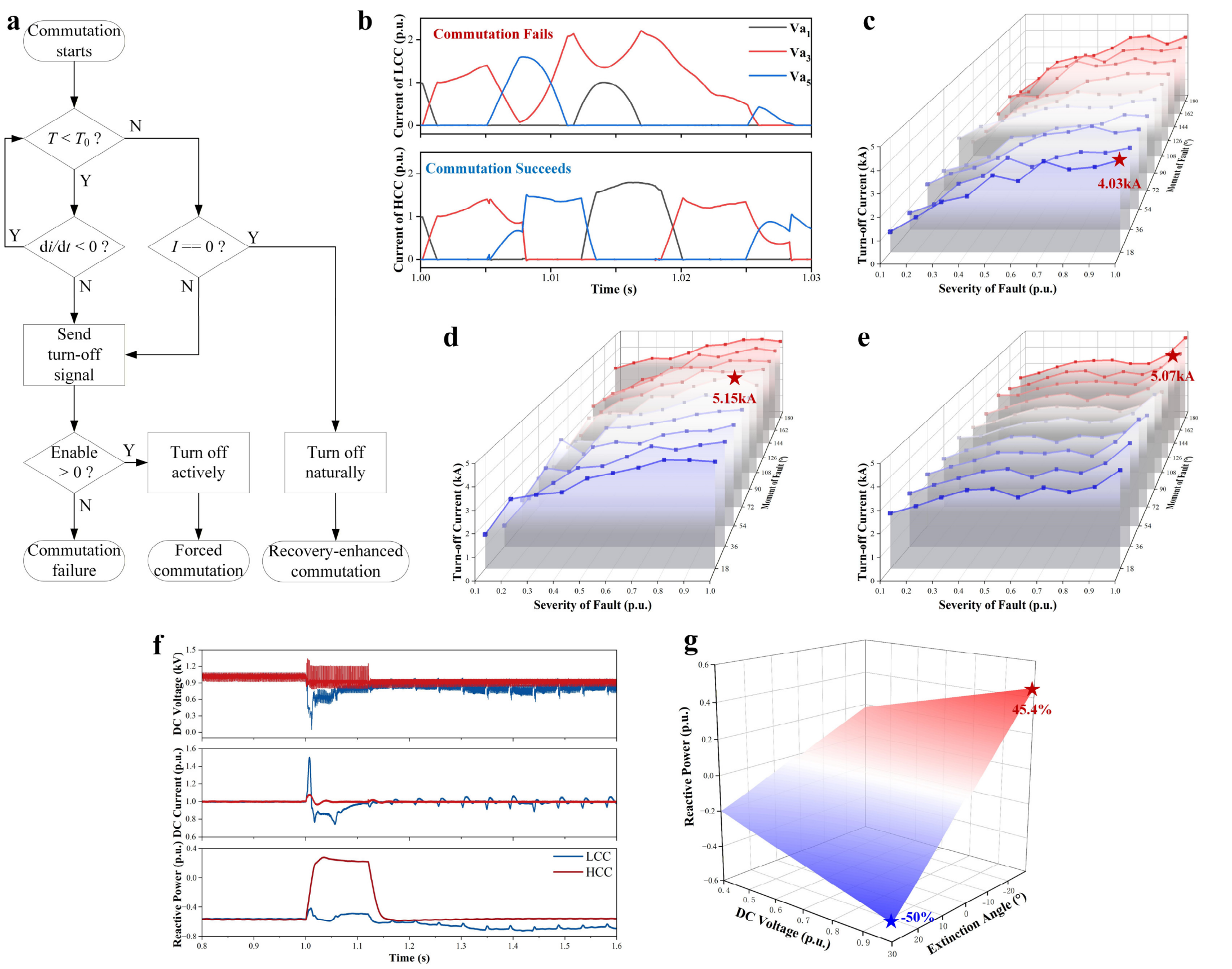

Fig. 4 Control flowchart and system operation characteristics of HCC. **a** Control flowchart. **b** Commutation of LCC and HCC during AC fault. **c** Turn-off current requirement under single-phase ground fault. **d** Turn-off current requirement under two-phase ground fault. **e** Turn-off current requirement under three-phase ground fault. **f** Performance of HCC and LCC during voltage sags. **g** Reactive power support during AC voltage oscillation.

stability in critical carbon-neutral applications, such as offshore wind power transmission, where such dynamic grid support is essential.”

Comment 5: *Clarification of HCC illustration*

In Figure 5(d), HCC is illustrated as a combination of SCR and IGBT. This raises questions regarding how higher capacity is achieved in practice, as the same current would flow through the IGBT as much as flow through SCR. It would be helpful for the authors to clarify whether the figure accurately reflects the intended structure, or whether it may have been oversimplified or misinterpreted. If the figure is correct, the illustrated HCC, it seems improved LCC rather than VSC by improved commutation characteristics. Can the authors elaborate further on this aspect?

We sincerely thank the reviewer for this insightful observation regarding Fig. 5d and the conceptual positioning of the HCC topology. The comment allows us to clarify

two crucial technical aspects.

First, we acknowledge that the use of "IGBT" in the original figure could indeed lead to misunderstandings. In the actual HCC implementation, the fully controlled semiconductor device employed is an **IGCT**, not an IGBT. This is a critical distinction because the IGCT, building upon the thyristor technology, possesses a **short-circuit current capacity equivalent to that of a conventional SCR**. This inherent characteristic is fundamental to achieving the high current-carrying capability of the HCC, ensuring that the current path through the IGCT can handle the same high-power levels as the SCR/LCC path, which is a prerequisite for high-capacity HVDC applications.

Second, the reviewer's interpretation that the HCC can be viewed as "improved LCC" is indeed accurate and aligns perfectly with our core concept. The fundamental innovation of the HCC lies in its strategic enhancement of the classic LCC structure. By replacing a portion of the SCRs in the conventional twelve-pulse bridge with IGCTs, we inject full-control capability directly into the LCC platform. This architectural choice is precisely what enables the HCC to **inherit the high capacity and low-loss advantages of LCC** while simultaneously acquiring the ability to **actively commutate current, thereby achieving complete immunity to commutation failures**.

As the reviewer astutely points out, this is an improvement on the LCC's commutation characteristics. Consequently, the HCC not only solves the LCC's primary weakness. but also **inherits key VSC attributes**, specifically the ability to regulate the reactive power characteristics at the converter terminals. This results in a converter that occupies a unique position: it offers the **commutation failure immunity and reactive power control that LCC lacks**, while maintaining a **superior economic profile and lower losses compared with a full-VSC solution**.

We have revised Fig. 5d to accurately reflect the use of IGCTs and have expanded the **Discussion** to elaborate further on this conceptual framing, emphasizing that the HCC represents a strategic evolution of the proven LCC platform rather than a completely new VSC-type structure. We believe this clarification significantly strengthens the manuscript's narrative.

"...commutation principles. **HCC employs IGCTs as its primary semiconductor device, based on the LCC architecture. As a thyristor-based technology, IGCTs not only inherit the advantages of conventional thyristors (namely high current-carrying capacity and low conduction losses), but also incorporate the active turn-OFF capability typically associated with IGBTs.** Therefore, ..."

Comment 6: *Grid-forming and system-level features*

The manuscript suggests that HCC could enable offshore wind integration, as implied in Figure 5(d). However, the power system–level implications remain unclear. Specifically, it would be important to discuss whether and how HCC can provide grid-forming (GFM) capabilities, and to demonstrate its active/reactive power performance in a system-level context. Such analysis would complement the device-level discussion and underscore the practical value of the proposed technology. (e.g., LCC HVDC can not provide this function. How about HCC?).

We sincerely thank the reviewer for raising this critical point regarding the system-level implications and grid-forming (GFM) capabilities of the proposed HCC topology. This comment helps us better articulate the technology's position and value in modern power systems.

The reviewer is correct in noting that the HCC, by its fundamental operating principle, **does not provide full GFM capability in the same way as a VSC**. Its commutation process remains dependent on the voltage of the connected AC grid, similar to a traditional LCC. Therefore, it cannot independently establish and maintain a stable grid voltage and frequency in a network without any other rotating generation.

However, as we have now clarified in the revised ***Operational Characteristics of HCC System*** section, this does not preclude its highly valuable role in system-level applications such as offshore wind integration. The key advantage of HCC over a classic LCC lies in its **flexible and controllable reactive power capability**. While an LCC is a net consumer of reactive power and requires large external compensators, the HCC can actively regulate the reactive power exchange at its terminals. This allows it to:

- **Provide dynamic voltage support** to the connected AC grid, significantly enhancing voltage stability over an LCC.
- **Operate over a wide range of power factors**, enabling it to support the grid during contingencies and normal operation without the need for additional STATCOMs or SVCs.

Regarding offshore wind, the HCC is a superior alternative to LCC. It can efficiently transmit the bulk active power from remote wind farms while simultaneously providing the necessary reactive power support for the wind farm collection grid and the onshore connection point—a function that a pure LCC cannot perform. Its immunity to commutation failures makes it robust against AC faults, a critical reliability advantage.

In summary, the HCC occupies a unique and valuable middle ground: it offers **substantially enhanced grid-supporting functions, particularly flexible reactive**

power control, which LCC lacks, while maintaining a **more economical and efficient profile than the full-VSC solution** that would be required for full GFM capability. We have revised the manuscript to make this system-level value proposition and the distinction between grid-following and grid-forming roles much more explicit. We believe this clarification strengthens the paper by accurately positioning the HCC for specific, high-value applications where full GFM capability is not the primary requirement.

"Beyond immunity from commutation failures, the HCC exhibits a distinct advantage over the LCC in providing reactive power support to the grid during voltage sags. Fig. 4f compares the performance of an LCC and an HCC under a 0.5 p.u. AC voltage dip. The HCC, endowed with robust reactive power support, maintains relatively stable voltage and current profiles, in stark contrast to the LCC. Fig. 4g delineates the operational range of the HCC's reactive power support. By adjusting the extinction angle across different DC voltage levels, the HCC can flexibly switch between absorbing and supplying reactive power. A pivotal capability is demonstrated at 1 p.u. voltage with an extinction angle of −30°, where the HCC can supply reactive power support at up to 45.4% of its nominal capacity. This performance marks a significant breakthrough for maintaining stability in critical carbon-neutral applications, such as offshore wind power transmission, where such dynamic grid support is essential."

"However, it is essential to acknowledge the current limitations of the HCC technology. The primary constraint is reduced flexibility compared with a full MMC. Furthermore, the HCC operates as a grid-following converter with voltage-support capability, and the grid-forming capability of it is still under research. Consequently, its optimal current application is in high-capacity, point-to-point or multi-terminal transmission corridors, solving the commutation failure problem rather than meeting the high flexibility demand. Future work will focus on exploring partial modularization within the efficient HCC architecture and refining the associated control strategies."

Thanks for the valuable comments and for exchanging academic and engineering views with the reviewer. The manuscript is improved a lot through the academic discussions. The authors analyzed and discussed seriously all the comments and questions raised by the reviewer. The descriptions, explanations, and comprehensive analysis are deepened and added to improve the quality of the study, which we hope, has addressed the concerns of the reviewer.

Response to the Reviewer #4

We would like to thank the reviewer's constructive suggestions. We have responded diligently and meticulously, making extensive revisions addressing your concerns. If you have any further inquiries or require additional modifications, we would be happy to revise them. For ease of referencing, the reviewers' comments are reproduced in ***black*** and our responses are given in ***blue***. The revised portions are marked in ***red*** in the new manuscript and the response letter.

Overall Comment: *The paper introduces an interesting concept with practical applications; several aspects could benefit from further clarification and deeper analysis to enhance its technical rigor and impact.*

We sincerely thank the reviewer for this overall positive assessment of our work's practical relevance and for the constructive suggestions to further enhance its quality. We have carefully addressed all the points raised throughout the review process, and we believe the revisions have significantly strengthened the manuscript's technical rigor, clarity, and impact.

Comment 1: *The structure and flow of the paper could be improved for readability. For example, the results section should not appear right after the introduction; please follow the section order as per the guidelines.*

We sincerely thank the reviewer for this comment regarding the paper's structure. We have carefully reviewed the journal's formatting guidelines and confirm that the current manuscript structure fully complies with the required section order. The sequence of sections is organized as follows: Introduction, Results, Discussion, Methods, which aligns precisely with the journal's specified structure. We appreciate the reviewer's attention to this detail and confirm that the section ordering in our manuscript adheres to the journal's guidelines. Should there be any specific aspect of the structural flow that requires further clarification or adjustment, we would be happy to receive more detailed feedback. Here, we give the instructions provided by *Nature Communications*:

"The main text of an Article should **begin with a section headed Introduction** of referenced text that expands on the background of the work (some overlap with the abstract is acceptable), **followed by sections headed Results, Discussion (if appropriate) and Methods (if appropriate)**."

Comment 2: *The paper highlights novelty in hybrid operation modes and device connection topology. To strengthen this claim, could you add a clearer comparison with*

existing hybrid HVDC concepts (e.g., LCC/VSC hybrids, series/parallel topologies)? It would also help if you explained which limitations of those approaches the proposed HCC addresses for clear understanding of the readers.

The authors attach great importance to the reviewer's comments on the LCC-VSC hybrid structure and have conducted corresponding research.

The LCC-VSC series topology, shown in Fig. D, is fundamentally characterized by the summation of voltages on the DC side. This configuration connects a cost-effective, low-loss LCC, responsible for handling the primary high voltage and power, in series with a VSC that bears a portion of the voltage while providing crucial control capabilities. This achieves a balance between UHVDC power transmission and economic viability. Its most prominent feature is the use of the VSC as a "voltage supporter", which **actively maintains the DC voltage during faults in the receiving-end AC grid, thereby reducing the commutation failure rate of the LCC**. Additionally, the VSC provides enhanced functionalities such as dynamic reactive power support and oscillation damping for the system. **However, a significant drawback of this topology lies in its highly complex coordination control and protection configuration.** A fault in any converter station affects the entire system, and the VSC section must withstand the full DC fault current, **imposing stringent requirements on the overcurrent capability of its semiconductor devices.**

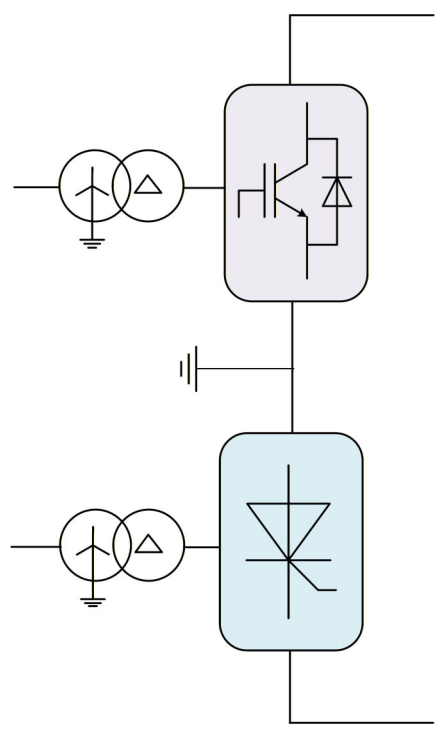

Fig. D The LCC-VSC series topology

The LCC-VSC parallel topology, shown in Fig. E, is characterized by the convergence of currents and the summation of power on the common DC bus. This structure connects both LCC and VSC converters to the same DC bus, enabling increased transmission capacity and complementary functionalities. Its greatest advantage lies in its **exceptional flexibility and redundancy**. The topology not only facilitates the straightforward formation of multi-terminal DC systems for flexible power dispatch, but also enables "N−1" operation, allowing the system to continue operating at a reduced capacity if a single converter station fails, thereby significantly enhancing power supply reliability. **The primary disadvantages of this topology are**

concentrated in DC voltage coordination control and fault management. It requires a highly coordinated control strategy to maintain voltage stability. Furthermore, during a DC-side short-circuit fault, the LCC continues to feed a substantial fault current, making fault clearance exceptionally difficult and typically **necessitating the use of costly high-speed DC circuit breakers.**

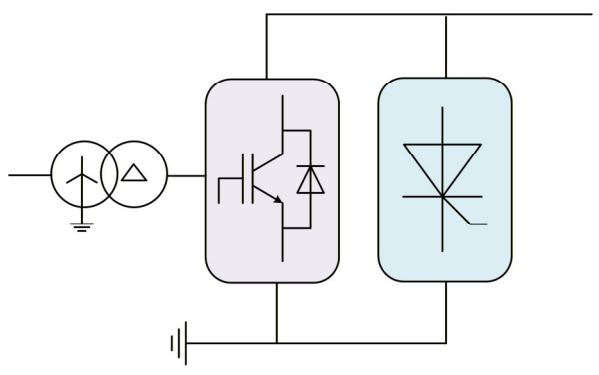

Fig. E The LCC-VSC series topology

Compared with LCC/VSC hybrid topologies, HCC achieves complete immunity to commutation failures by replacing a portion of its components, resulting in lower costs and eliminating the need for complex control and protection configurations. Furthermore, because IGCT devices possess the same short-circuit current capacity as thyristors, HCC does not require DC circuit breakers.

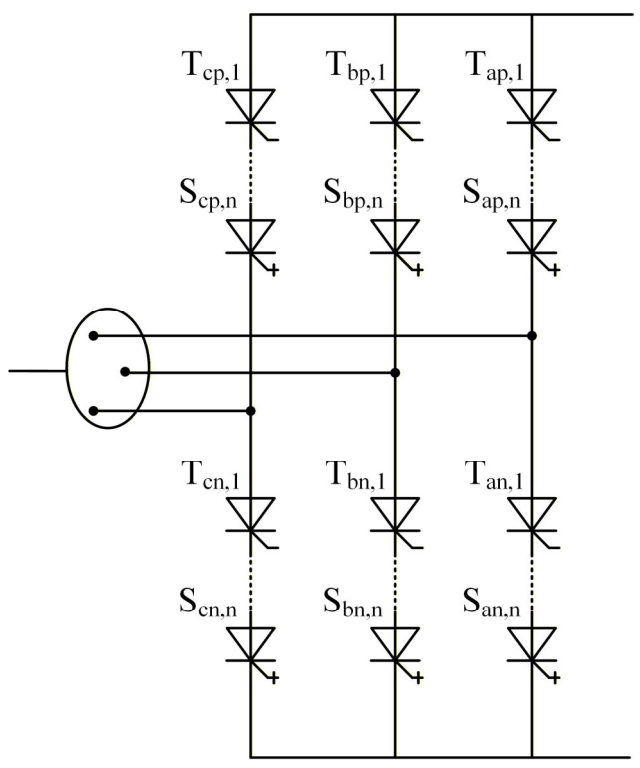

Fig. F The proposed HCC topology

We have revised the **Introduction** to include this comparison between HCC and LCC-VSC hybrid topologies. In the **Discussion**, the authors also provide an economic comparison between LCC, VSC, LCC-VSC hybrid, and HCC topologies.

"**The LCC-VSC hybrid topology can partially mitigate commutation failure issues, but its system-level architecture is inherently complex and costly. Specifically, the LCC-VSC hybrid solution requires two completely independent converter systems, with their associated control systems. This leads to challenges in terms of coordinated control, a larger physical footprint, and the necessity of DC circuit breakers for effective fault management.**"

Comment 3: *The paper introduces 'forced commutation' and 'recovery-enhanced commutation.' To improve clarity, it would help to include a more detailed mathematical framework or flowcharts showing their operation under different fault conditions and*

to explain more clearly how the two modes differ.

We sincerely thank the reviewer for the constructive suggestion regarding the clarification of the forced and recovery-enhanced commutation modes. In direct response to this comment, we have added a detailed operational flowchart in the revised manuscript, specifically in the **Results** section (part D).

The newly introduced flowchart systematically illustrates the decision-making logic and execution sequence for selecting between the two commutation modes under varying fault conditions. Its core logic is parsed as follows:

1. **Initial Condition Check:** The process begins by determining whether the system is within the "commutation window", which means the commutation has started. This is the foundational temporal criterion for mode selection.

2. **Pathway for Recovery-Enhanced Commutation:** If the system is not within the commutation window and the measured bridge arm current is zero, the system enters the recovery-enhanced commutation mode. This mode is designed for scenarios where the fault allows the current to naturally decay to zero.

3. **Pathway for Forced Commutation or Failure:** Two primary conditions trigger the evaluation for forced commutation:

- The system is within the commutation window and the current derivative ($\mathrm{d}i/\mathrm{d}t$) is detected to be positive.
- The system is not within the commutation window, but the bridge arm current is not zero.

Upon satisfying either condition, the final step depends on the status of the active turn-off enable signal:

- If the enable signal is 1 (active), the system successfully engages the forced commutation mode, using the IGCTs' full-control capability to actively interrupt the current.
- If the enable signal is 0 (inactive), the system cannot execute active turn-off, resulting in commutation failure.

This flowchart and its accompanying description now provide a clear, unambiguous mathematical and logical framework. It distinctly differentiates how the HCC dynamically selects its commutation strategy based on real-time electrical measurements (current, $\mathrm{d}i/\mathrm{d}t$) and timing (commutation window), effectively demonstrating the operational difference between the two modes and their respective roles in preventing commutation failure.

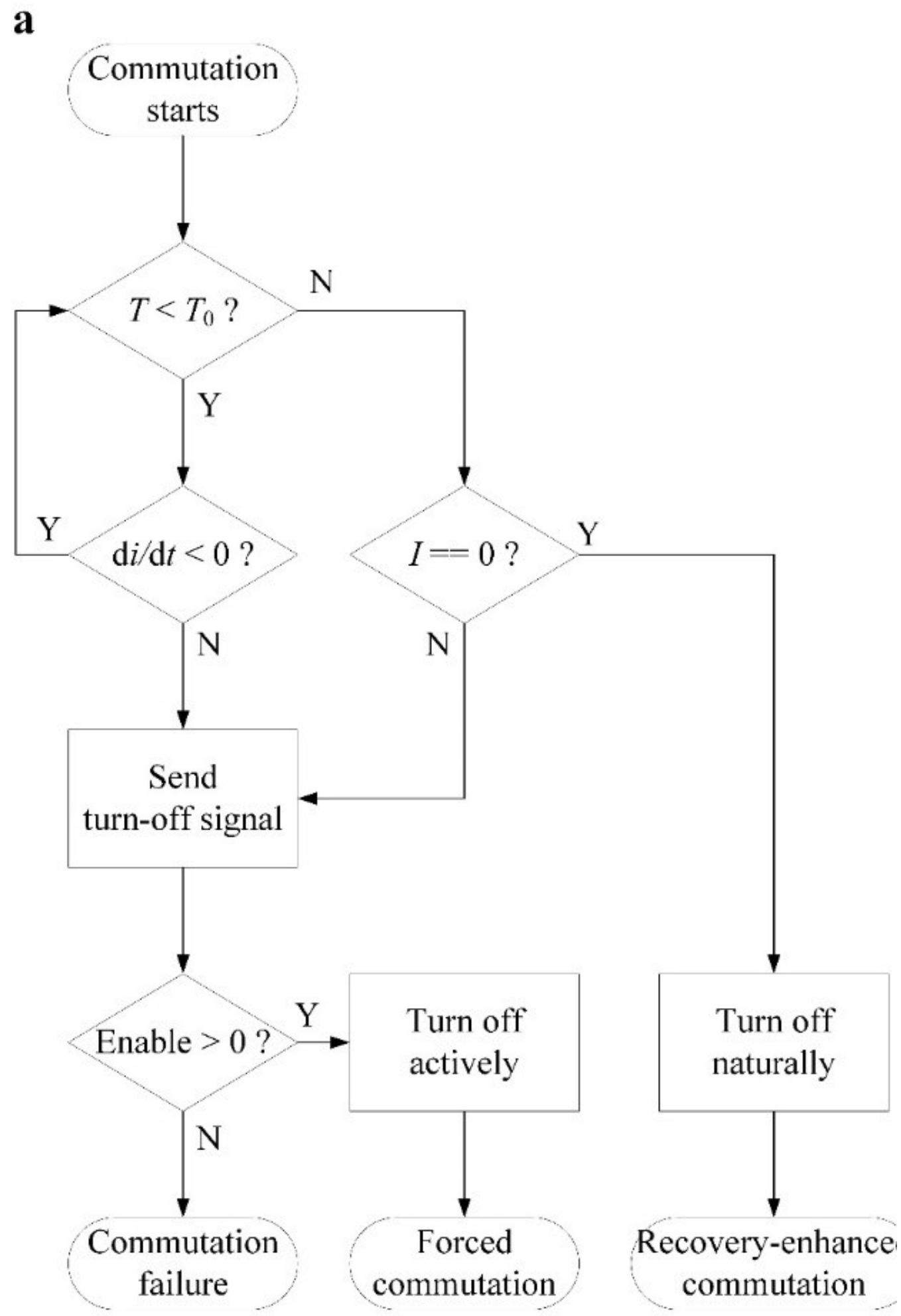

Fig. 4a Control flowchart of HCC

Comment 4: *The mathematical equations' variables should be clearly defined to easily grasp the idea.*

We thank the reviewer for this comment regarding the definition of mathematical variables. In response to this point, we have ensured that all variables used in the mathematical equations throughout the manuscript are clearly defined. A complete and dedicated nomenclature section has been inserted at the beginning of the paper, listing each variable along with its description and units, ensuring readers can easily grasp the concepts presented in the formulations. We believe this addition enhances the clarity and accessibility of the manuscript's technical content.

"

R_a, R_b	**Static voltage balancing resistance (Ω)**
C_s, C_t	**Dynamic voltage snubber capacitance (µF)**
R_s, R_t	**Dynamic voltage threshold snubber resistance (Ω)**

R_{mov}	Equivalent resistance of MOV_t (Ω)
I_{main}	Main-loop current (A)
I_1	Current of dynamic voltage threshold circuit (A)
I_2	Current of overvoltage clamping MOV (A)
U	Voltage over the IGCT (V)
U_C	Voltage over snubber capacitor (V)
U_{mov}	Voltage of MOV_t (V)
U_{ref}	Reference voltage of MOV (V)
U_{res}	Residual voltage of MOV (V)
t_1	Time of voltage rising to U_{res} (s)
t_{res}	Time of steep wave impulse current front (s)
K	Nonlinear coefficient of MOV
P_{mov}	Designed rated power of MOV (W)
E_{J1}, E_{J2}	Electric fields within the depletion layers (V/cm)
N_{Nb}	Doping concentration of the N base region (cm^{-3})
ε_{Si}	Dielectric constant of silicon (F/cm^2)
W_{d1}, W_{d2}	Depletion layers of J_1 and J_2 (cm)
v_{sat}	Saturation velocity (cm/s)
W_{Nb}	Width of the N base region (cm)
p_{x1}, p_{x2}	Boundary carrier concentrations (cm^{-3})
F	Feedback constant ($V\cdot cm^3$)
V_{d1}, V_{d2}	Voltages of depletion layers (V)
A	Total active area (cm^2)
q	Electric charge (C)
D	Ambipolar diffusivity (cm^2/s)
D_n	Electron diffusivity (cm^2/s)

D_p **Hole diffusivity (cm^2/s)**

I_{n1}, I_{p1} **Electron and hole current at x_1 edge of undepleted drift region (A)**

"

Comment 5: *The paper mentions carbon neutrality and reduced footprint. It would be helpful to include an economic analysis comparing HCC-HVDC with LCC- and VSC-HVDC and to quantify the carbon benefits with clear metrics.*

We sincerely thank the reviewer for this valuable suggestion to enhance the paper's practical and environmental impact. We agree that a quantified economic and carbon benefit analysis is crucial for objectively demonstrating the advantages of HCC-HVDC.

In direct response to this comment, we have added analysis in the **Discussion** of the revised manuscript. This part provides a systematic comparison based on established project metrics.

"**The economic argument for HCC is compelling, particularly when viewed through the lens of lifecycle cost and its role in enabling carbon neutrality. A comparative analysis is summarized in Table 1. For long-distance, large-scale renewable energy transmission projects, such as delivering power from remote "sand-gobi-desert" renewable bases in China or similar settings worldwide, the cost per megawatt-kilometer is a decisive factor. The economic profile of the HCC, which approaches that of LCCs while offering VSC-like stability, significantly reduces the levelized cost of electricity for clean energy. By providing a reliable, high-capacity corridor without the risk of commutation-failure-induced outages, the HCC enhances grid utilization and reduces the need for redundant backup infrastructure. This improved efficiency and reliability directly translate into lower system-wide costs and accelerated deployment of renewable energy, thereby delivering substantial carbon mitigation benefits over the project's lifetime.**"

Table 1 Comparative Analysis of HVDC Converter Technologies

Aspect	LCC	VSC(MMC)	LCC-VSC Hybrid	HCC (This work)
Capital Cost	Low	High	Very High	Low (close to LCC)
Operational Losses	Low	High	Medium-High	Low (inherited from LCC)
Commutation Failure Risk	High	None	Low	None (Active immunity)
Reactive Power Support	Consumer (requires external	Full, independent control	Provided by VSC part	Controllable support

	compensation)			
System Complexity	Low	Medium	Very high	Low
Key Enabler for Renewables	Limited by stability concerns	Excellent but costly	Effective but expensive	High-capacity, stable & cost-effective

We have incorporated a summary table comparing key metrics. We believe this addition provides the clear, metric-driven comparison requested, solidifying the argument for HCC's role in sustainable energy infrastructure.

Comment 6: *Improve the English and correct minor grammatical errors and formatting inconsistencies.*

We thank the reviewer for pointing out the need for language and formatting improvements. In response to this comment, we have conducted a thorough proofreading and language polishing of the entire manuscript. This process included correcting grammatical errors, improving sentence clarity and flow, and ensuring consistency in terminology, abbreviations, and formatting (e.g., figures, tables, and references) throughout the paper. We believe these revisions have significantly enhanced the readability and professional presentation of the manuscript.

Comment 7: *Do elaborate on the limitations and potential improvements of the proposed HCC method.*

We sincerely thank the reviewer for raising this important point. A balanced discussion of limitations is crucial for a complete and objective assessment of any new technology. We have expanded the **Discussion** section in the revised manuscript to explicitly address the limitations and outline clear pathways for future improvements to the proposed HCC method.

"**However, it is essential to acknowledge the current limitations of the HCC technology. The primary constraint is reduced flexibility compared with a full MMC. Furthermore, the HCC operates as a grid-following converter and lacks the full grid-forming capability required to establish the network voltage and frequency autonomously. Consequently, its optimal current application is in high-capacity, point-to-point transmission corridors, rather than in multi-terminal HVDC grids demanding high flexibility. Future work will focus on exploring partial modularization within the efficient HCC architecture and refining the associated control strategies.**"

We have integrated this analysis into the manuscript. We believe this honest appraisal of the technology's current boundaries, coupled with a vision for its evolution,

strengthens the scholarly contribution by providing a realistic and forward-looking perspective.

Thanks for the valuable comments and for exchanging academic and views with the reviewer. The manuscript is improved a lot through the academic discussions. The authors analyzed and discussed seriously all the comments and questions raised by the reviewer. The descriptions, explanations, and comprehensive analysis are deepened and added to improve the quality of the study, which we hope, has addressed the concerns of the reviewer.

Response Letter (NCOMMS-25-43025B)

Dear Reviewers,

We are very grateful to you and the reviewers for his/her appreciation of this work and the time invested in reviewing the manuscript (ID: **NCOMMS-25-43025-T** and **NCOMMS-25-43025A**). We have amended the relevant parts in the manuscript according to the suggestions, which are all denoted by the red font. All of the questions were answered below item by item in this letter.

For ease of referencing, the reviewers' comments are reproduced in ***black*** and our responses are given in ***blue***. The revised portions are marked in ***red*** in the new manuscript and the response letter.

In this revision, the authors have focused on refining the subsection titled "**HCC System Operational Characteristics**" to provide a clearer description of the **HCC control architecture**. Furthermore, time-domain waveforms of the AC voltage have been added in the sections discussing **commutation failure immunity** and **reactive power support**, in order to explicitly define the fault input conditions. In the **Discussion** section, the distinct advantages of applying HCC in **offshore** and **onshore** scenarios, respectively, have been clarified. Additionally, the **comparison between different converter technologies** in Table 1 has been **quantified** with specific references provided. The associated data and code are all provided with the paper.

With the help of reviewers, we have provided a broader view of the HCC system and its application. The manuscript has been substantially improved in terms of academic rigor, clarity of presentation, and completeness of argumentation. Thanks for the attention of our paper. We look forward to hearing from you.

Best regards,

Zhanqing Yu, Professor
Department of Electrical Engineering, Tsinghua University.

Rong Zeng, Professor
Department of Electrical Engineering, Tsinghua University.

Response to the Reviewer #2

Overall Comment: *Thank you for the authors' response and revisions. All comments have been properly addressed, and the reviewer has no further comments.*

Thank you for your positive feedback and for confirming that all comments have been properly addressed.

We sincerely appreciate the time and expertise you dedicated to reviewing our manuscript. Your insightful comments and suggestions were valuable in improving the quality of our work.

Response to the Reviewer #3

We would like to thank the reviewer's constructive suggestions again. We have responded diligently and meticulously, making extensive revisions addressing your concerns. If you have any further inquiries or require additional modifications, we would be happy to revise them. For ease of referencing, the reviewers' comments are reproduced in ***black*** and our responses are given in ***blue***. The revised portions are marked in ***red*** in the new manuscript and the response letter.

Overall Comment: *The reviewer appreciates the authors' previous responses. This reviewer still has several key concerns of HCC for power transmission system as this paper suggested.*

Thank you for your continued engagement with our manuscript and for your acknowledgment of our previous responses. We also sincerely appreciate you taking the time to raise concerns regarding the application of HCC in power transmission systems, as suggested in the paper.

We have carefully considered each of the points you highlighted. In the response below, we provide a point-by-point reply to your valuable comments and have correspondingly revised the manuscript to address your suggestions.

Comment 1: *Figure 6 should be revised for clarity. As noted in the authors' response, an HCC-based HVDC system cannot access offshore renewable energy sources without grid-forming resources, either from generators or converters. However, the current representation of Fig. 6 may suggest that the HCC can directly interface with offshore renewables, which introduces ambiguity. In particular, since the offshore MMC is replaced by the HCC, Fig. 6(d) may be misleading and should be clarified or modified accordingly.*

We sincerely thank the reviewer for your careful and thorough review of the revised manuscript and the response letter, as well as for your valuable insights regarding the application of HCC in the offshore context.

While the authors did state in the previous response that HCC "cannot independently establish and maintain a stable grid voltage and frequency in a network without any other rotating generation," it is important to clarify that **this statement was intended to highlight HCC's relatively weaker grid-forming capabilities compared to MMC, rather than to imply that it is unsuitable for offshore applications.** Furthermore, in the same response, the authors dedicated significant space to

elaborating on the distinctive features of HCC. These include its ability to provide **dynamic voltage support** (compared to LCC), a **wider operating power factor range** (compared to LCC), and **greater cost-effectiveness** (compared to MMC). These characteristics collectively make HCC a technically viable and economically advantageous option for offshore renewable energy integration.

The authors have added this elaboration in the manuscript to strengthen the argument for the applicability of HCC in offshore scenarios:

"**When applied offshore, HCC's dynamic voltage support capability and wide power factor operation range are sufficient to enable the integration of renewable energy, significantly reducing both cost and physical footprint.**"

The supplementary discussion on HCC's dynamic voltage support above has laid the groundwork for its application in offshore wind farms. Meanwhile, existing research on voltage[1] and frequency[2] control of doubly-fed induction generators (DFIG) in offshore wind farms further optimizes the operating conditions for HCC in such scenarios. Additionally, **equipping HCC with STATCOM and achieving coordinated dispatch and control between them** to jointly provide voltage support represents a more feasible solution, which is currently a focus of research in our group.

[1] Dawei Xiang, Li Ran, J. R. Bumby, P. J. Tavner and S. Yang. Coordinated Control of an HVDC Link and Doubly Fed Induction Generators in a Large Offshore Wind Farm. *IEEE Transactions on Power Delivery*, **21**, **1**, 463-471 (2006).

[2] R. Li, S. Bozhko and G. Asher. Frequency Control Design for Offshore Wind Farm Grid With LCC-HVDC Link Connection. *IEEE Transactions on Power Electronics*, **23**, **3**, 1085-1092 (2008).

Thank the reviewer for the valuable comment. According to the comment of the reviewer, related analysis and descriptions have been added to the revised manuscript for a better understanding to the readers.

Comment 2: *In addition, it is unclear why no harmonic filter is shown for the HCC–HVDC system. Does the HCC exhibit harmonic performance comparable to that of an MMC, or would a harmonic filter still be required? Elaborating on this aspect will be beneficial for power system community.*

We sincerely thank the reviewer for your comment regarding the HCC harmonic filter, which has helped us optimize the description of the HCC system in the manuscript.

Since HCC is optimized based on the fundamental architecture of LCC, its operating region still encompasses that of LCC itself. In other words, HCC expands the operating domain **from two-quadrant operation**, which can only absorb reactive power, as shown in Fig. I(a), **to four-quadrant operation**, which can both absorb and

supply reactive power, as shown in Fig. I(b).

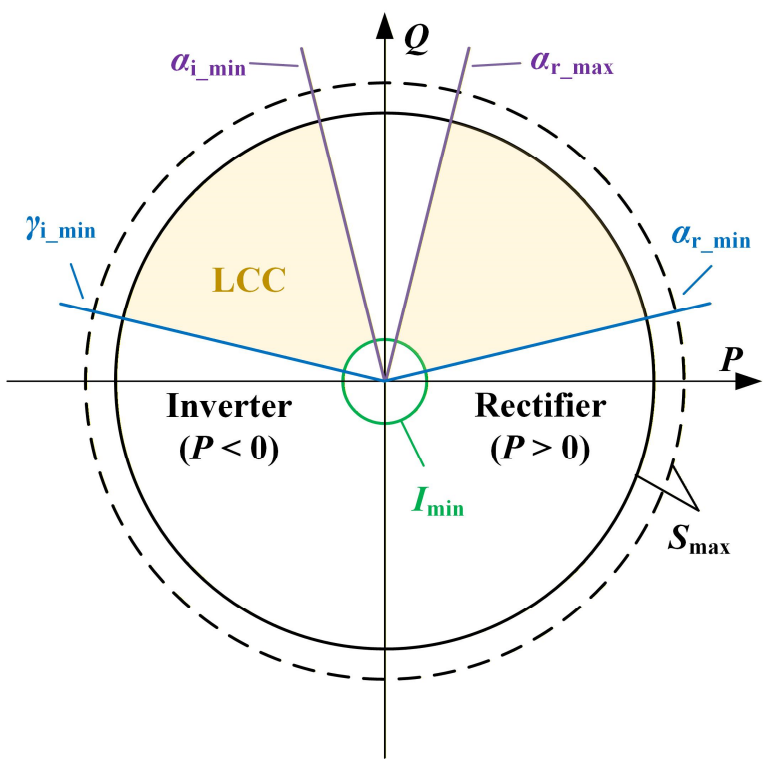

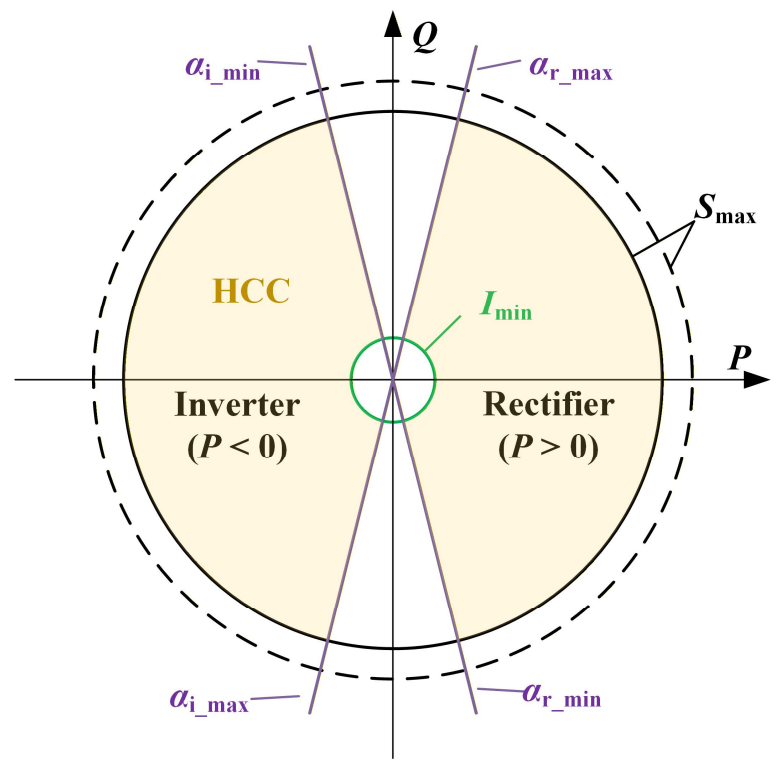

(a) (b)

Fig. I Comparison of operating regions between HCC and LCC: (a) LCC; (b) HCC

At different operating points, **the harmonics of the HCC converter depend primarily on its extinction angle**. When HCC operates under the same conditions as LCC (e.g., with an extinction angle of 17°), the harmonic content is high, and filters must be put into operation to eliminate harmonics. However, when HCC operates in the extended region (e.g., with an extinction angle of 0°), the harmonic content decreases. In this case, not all filters are required for harmonic elimination, and the surplus filters can be used together with the HCC itself to provide reactive power support to the grid. In summary, **filters are indispensable to ensure that HCC remains compatible with LCC operating conditions, but their configuration is more flexible**.

Regarding the filter configuration, the following revisions have been made in the manuscript, and Fig. 6(d) has been revised accordingly:

"When deployed onshore, its commutation failure immunity and fault ride-through capability help avoid interruptions in power transmission. Moreover, due to its extended operating range, HCC can utilize surplus filter banks to provide reactive power support to the grid, further strengthening its application advantages."

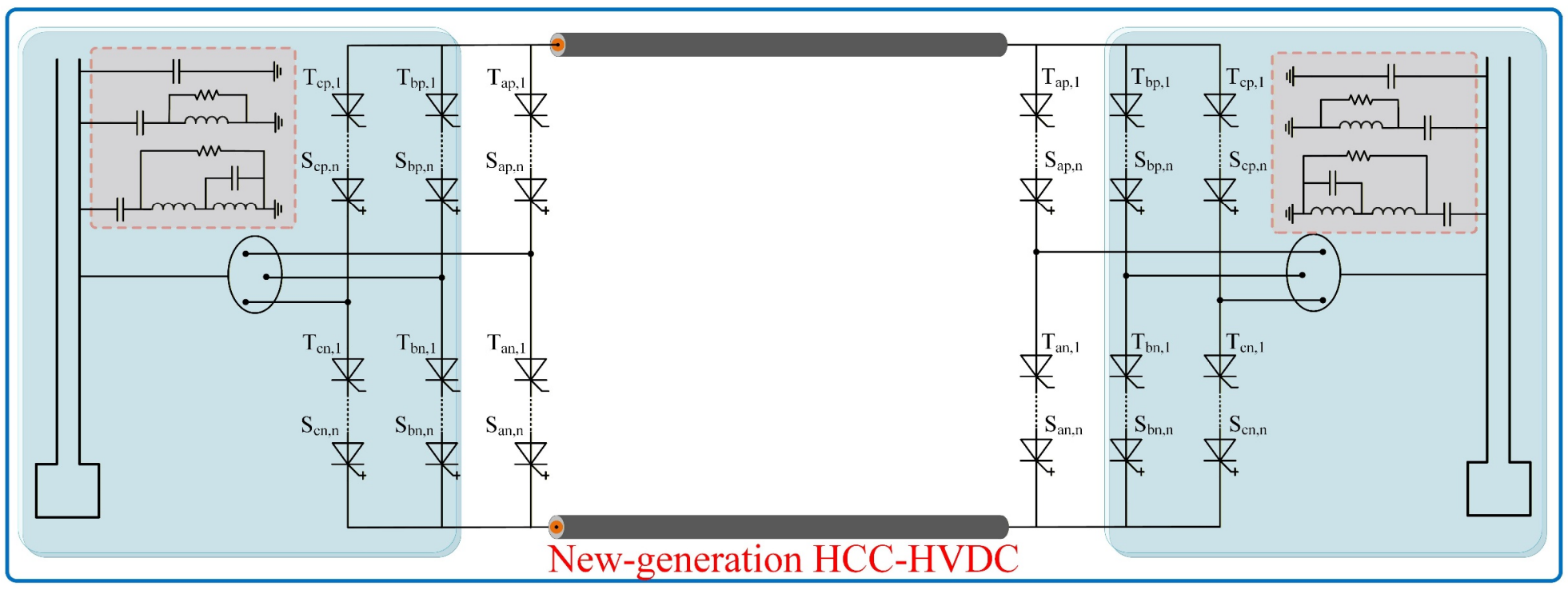

Fig.6 d Offshore and onshore power conversion based on the HCC.

We sincerely thank the reviewer again for your careful review and valuable suggestion so we can clarify the operating characteristics of HCC. We hope it can be helpful and interesting to communities working on power system.

Comment 3: *In Figure 4, the AC voltage waveform is not shown. Including the AC voltage signal, in time domain rather than static plotting as currently does, would improve clarity. Furthermore, the manuscript does not clearly explain how reactive power is controlled during AC voltage interruption. The control mechanisms and schemes should be explicitly described. The authors are encouraged to provide time-domain results for a three-phase fault case as well to highlight its reactive power support capability as it is one of main advantages of HCC HVDC compared to LCC. It would also be helpful to clarify whether the HCC can provide reactive power support under normal operating conditions.*

Thank you for your clear and detailed suggestions. The authors have discussed and carefully revised the manuscript to address all the points you raised.

Regarding the inclusion of AC voltage waveforms in the time domain: As suggested, the authors have updated Fig. 4c and 4g accordingly. Specifically, in the revised Fig. 4c, the original single-phase-to-ground fault case was replaced with a three-phase-to-ground fault to provide a clearer contrast. The results distinctly show that after the three-phase voltage drops completely to zero, LCC fails to commutate properly, whereas HCC maintains orderly commutation through active turn-off. Furthermore, HCC demonstrates faster recovery after fault clearance. For the revised Fig. 4g, the authors note that this simulation was conducted under a three-phase fault condition with a voltage dip of 0.5 p.u.. Following your advice, we have added the corresponding three-phase AC voltage waveforms in the time domain to this figure. Corresponding descriptions have been added to the manuscript as follows:

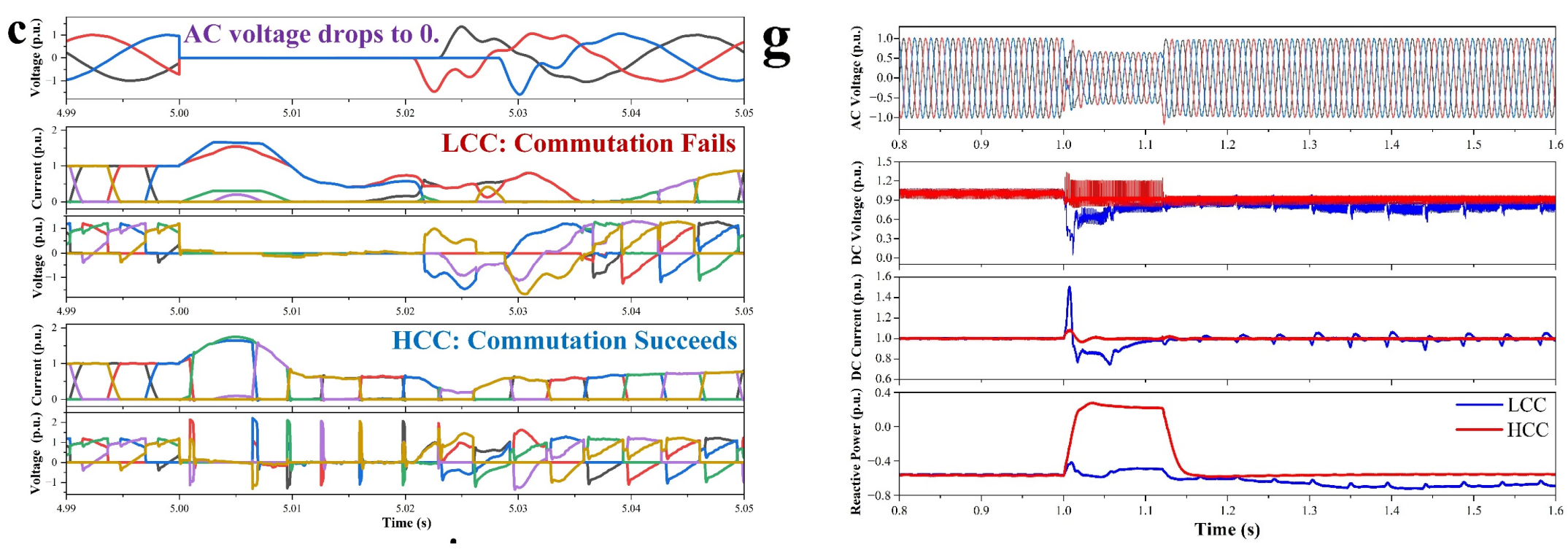

Fig.4 c Commutation of LCC and HCC during AC fault. g Performance of HCC and LCC during three-phase voltage sags.

"**The system-level control structure of HCC** provides effective immunity against commutation failures across all AC system fault conditions. A representative commutation failure mitigation process during a three-phase-to-ground fault is depicted in Fig. 4c. **The data indicate that due to its inability to mitigate commutation failures, LCC experiences successive commutation failures after the three-phase voltage drops to zero. Once all bridge arms conduct sequentially, orderly commutation cannot be maintained, and normal operation cannot be resumed even after the AC voltage recovers. In contrast, HCC achieves orderly commutation by actively turning off the devices to construct commutation voltage**".

Regarding the reactive power control scheme during AC voltage dips and its capability under normal conditions: The authors have added detailed explanations in the manuscript to clarify the control mechanisms. The added text describes both the method for reactive power control during faults and the capability for reactive power support during normal operation:

"**Specifically, two scenarios are considered regarding reactive power control. During an AC voltage dip, the reactive power reference is adjusted according to the severity of the dip—the deeper the voltage drops, the higher the reactive power reference. This predefined correspondence, in the form of characteristic curves, is stored in the control system to enable automatic reactive power adjustment in response to voltage sags. During normal operation, if the system requires reactive power support, the reactive power reference can be manually specified. The corresponding extinction angle is then calculated based on the relationship between reactive power and the extinction angle and fed into the control loop, thereby enabling the converter to provide the necessary reactive power support to the system**".

Comment 4: *As pointed out in the reviewer's previous comments and in relation to the above issues, the system-wide control structure of the HCC–HVDC system should be provided. Providing the corresponding control structure would significantly improve clarity. This should include system-wide control structures such as the outer control loops (Power & Reactive power), current controllers, and the synchronization mechanism (e.g., PLL or alternative methods). Otherwise, it is remained unclear how it works for transmission system.*

We sincerely thank the reviewer for this comment. In response, we have provided a detailed description of the overall control framework for the HCC system in the revised manuscript (e.g., in Fig. 4). The control structure is comprised of **turn-ON**

control and **turn-OFF control**.

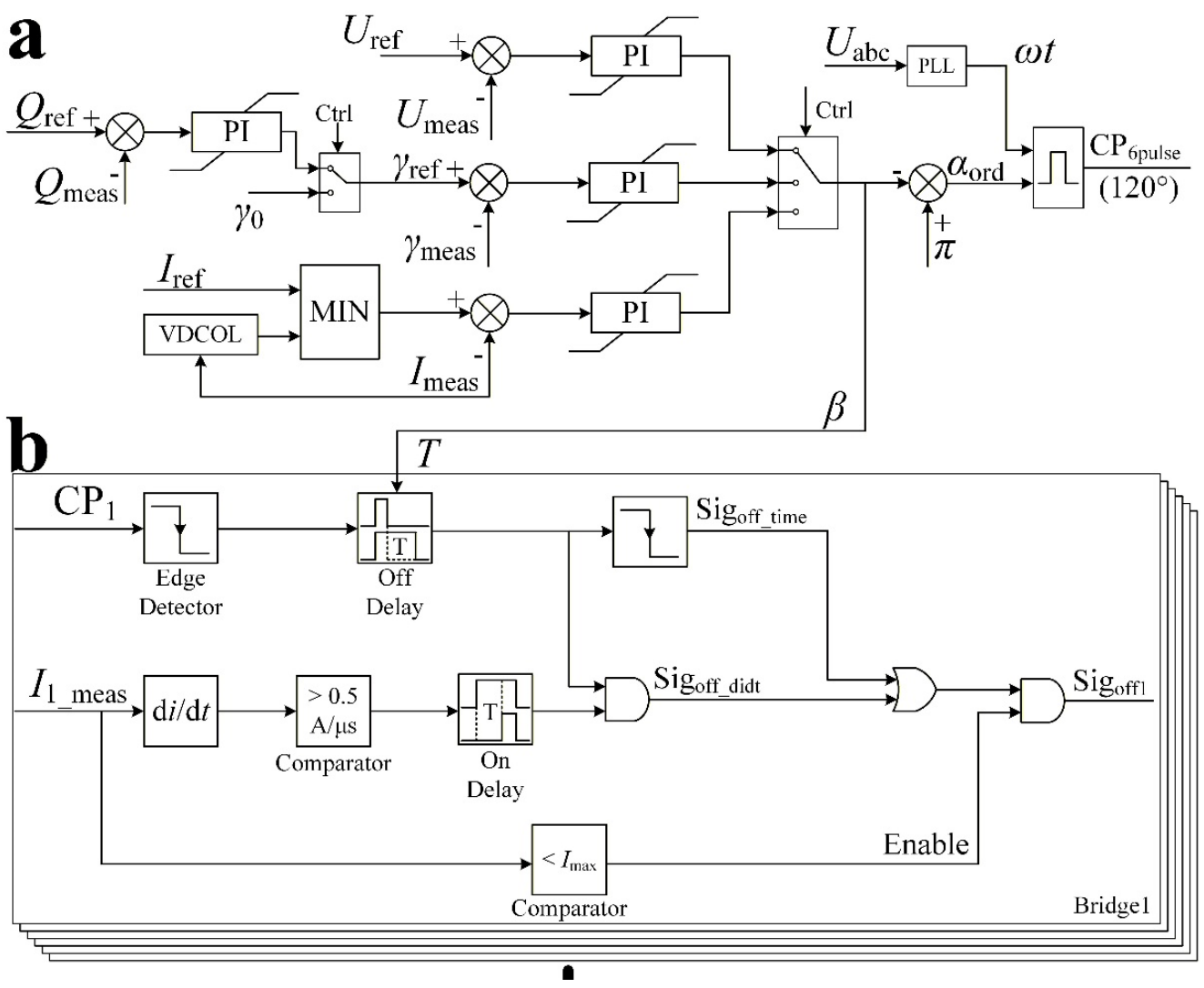

Fig. 4 a Turn-ON control structure. b Turn-OFF control structure.

The **turn-ON control** largely follows conventional LCC technology: the rectifier employs constant DC current control, while the inverter employs either constant DC voltage control or constant extinction angle control. The firing angle derived from these controllers is then used by a Phase-Locked Loop (PLL) to generate the firing signals for each valve bridge. A key difference in the HCC's turn-ON control is that the extinction angle reference can optionally be generated based on reactive power demand, enabling HCC to regulate reactive power.

The **turn-OFF control** is a new addition in HCC compared to conventional LCC. It includes both **timed turn-OFF** (which ensures reliable commutation under all conditions) and **di/dt-based turn-OFF** (which reduces turn-off stress). Furthermore, an overcurrent protection scheme is integrated into the control system to prevent the RB-IGCTs from turning off currents beyond their capability.

This clarification on the system-level control structure significantly improves the explanation of how the HCC-HVDC system operates within a transmission network.

Regarding the system control structure, the following content has been added to the manuscript, and Fig. 4 has been revised accordingly:

"The system-level control structure of HCC consists of two parts: turn-ON control and turn-OFF control. The turn-ON control follows the conventional LCC system control structure, which includes constant voltage mode, constant current mode, and constant extinction angle mode, like shown in Fig.4a. Generally, the rectifier adopts constant current control, while the inverter adopts constant extinction angle control. These control methods ultimately generate the nominal firing angle (α) required for the turn-ON signals, which are then used by a phase-

locked loop (PLL) to produce six-pulse firing signals. In addition to the conventional turn-ON control, HCC introduces an additional mode for regulating the extinction angle based on reactive power demand, thereby enabling reactive power control."

"**The turn-OFF control is a unique control module that distinguishes HCC from LCC, with its control actions precisely targeted at each bridge arm, like shown in Fig.4b. First, a commutation window with a duration of *β* is generated at the falling edge of the turn-ON signal, and a turn-OFF command is issued to the bridge arm at the end of this window. If a positive di/dt is detected during the commutation window, a turn-OFF command is issued in advance at that moment to prevent further development of fault current. Furthermore, the HCC system incorporates an overcurrent protection strategy to avoid damage caused by turning off currents exceeding the capability limits of the RB-IGCT.**"

Thank you again for your constructive comments and for exchanging academic and engineering views. The authors analyzed and discussed seriously all the comments and questions raised by the reviewer including this revision and last revision. The authors believe these revisions have significantly improved the clarity and completeness of the presentation regarding HCC's application, control system and operating characteristics. We hope the work will be helpful and interesting to communities working on power system and high-power transmission.

Response to the Reviewer #4

We would like to thank the reviewer's constructive suggestions. We have responded diligently and meticulously, making extensive revisions addressing your concerns. If you have any further inquiries or require additional modifications, we would be happy to revise them. For ease of referencing, the reviewers' comments are reproduced in ***black*** and our responses are given in ***blue***. The revised portions are marked in ***red*** in the new manuscript and the response letter.

Overall Comment: *The author has thoroughly answered questions in the revised manuscript. However, to improve the content, it needs to be arranged the format according to the journal guidelines.*

We sincerely thank the reviewer for your positive feedback on our revisions and for your additional suggestion regarding the manuscript format. We appreciate the time and expertise you have dedicated to reviewing our work. In response to your comment, we have carefully revised the manuscript to ensure full compliance with *Nature Communications* formatting guidelines. We have also taken this opportunity to perform a thorough proofreading to polish the language throughout the text.

Comment 1: *The manuscript does not fully follow the journal's sectioning guidelines. Specifically, the Introduction section is not clearly labeled after the Abstract, and the Nomenclature section is also missing an explicit heading. This lack of proper section titles may cause confusion for readers. Please revise the manuscript to ensure that all sections are clearly titled and structured in accordance with the journal's formatting guidelines.*

Thank you for your feedback regarding the manuscript's structure and formatting. We have revised the manuscript to fully address your comment. The **Introduction** and **Nomenclature** sections are now clearly labeled with proper headings. Furthermore, the entire document has been reformatted according to *Nature Communications* guidelines, including adjustment to a single-column layout, double-line spacing, and unjustified text alignment and so on, which we hope, providing a better understanding to the readers.

Comment 2: *The overall quality of English needs improvement. The authors are advised to revise the manuscript carefully and consider professional language editing to enhance clarity, grammar, and readability.*

The authors attach great importance to the reviewer's comments on improving the

English language of the manuscript. We have carefully revised the entire text to enhance its clarity, grammar, and readability. The language has been polished to be more professional and concise.

Thank you again for your valuable comments. We believe these revisions have further improved the clarity and presentation quality of the manuscript, which we hope, helping bring better readability to readers.

Response Letter (NCOMMS-25-43025C)

Dear Reviewers,

We are very grateful to you for the appreciation of this work and the time invested in reviewing the manuscript (ID: **NCOMMS-25-43025-T**, **NCOMMS-25-43025A** and **NCOMMS-25-43025B**).

With your help, we have provided a broader view of the Hybrid Commutated Converter system and its application. The manuscript has been substantially improved in terms of academic rigor, clarity of presentation, and completeness of argumentation. Thanks for the attention of our paper.

Best regards,

Zhanqing Yu, Professor
Department of Electrical Engineering, Tsinghua University.

Rong Zeng, Professor
Department of Electrical Engineering, Tsinghua University.

Response to the Reviewer #3

Overall Comment: *The authors addressed the comments.*

Thank you for your feedback and for confirming that all comments have been properly addressed.

We sincerely appreciate the time and expertise you dedicated to reviewing our manuscript. Your insightful comments and suggestions were valuable in improving the quality of our work.
