# Supplementary material for: High-power hybrid commutated converter for carbon-neutral power transmission
Source: Nat Commun. 2026 Jul 22;17:8912. doi: 10.1038/s41467-026-75691-5 (PMC13503722; doi:10.1038/s41467-026-75691-5)
Supplement: Supplementary file 4 [file 41467_2026_75691_MOESM4_ESM.zip › Instruction.docx]

**INSTRUCTION**

**Both models need to run in MATLAB/SIMULINK whose version higher than 2022a.**

**1. Forced Commutation Principle**

**Model composition:** As shown in Fig. 2 of the manuscript, the device-circuit coupling electromagnetic simulation model includes an equivalent DC source, a hybrid bridge, a thyristor bridge, and an equivalent AC source. The equivalent sources adopt LC oscillation to fit the voltage and current in the actual circuit, and the IGCT in the hybrid bridge adopts a physics-based compact model.

**Method of application:**

1. Before conducting simulation research, run the "Simulation_Forced.m" configuration file to complete the parameter definitions in MATLAB.

2. Set the external circuit parameters in "Simulation_Forced.slx", including all capacitors and inductors, as well as the snubber parameters of the devices.

3. Run the "Simulation_Forced.slx" model to obtain the desired simulation results in SIMULINK.

**Points for attention:**

1. Due to the fact that this condition studies the forced commutation principle, it is not important whether the thyristor has reverse recovery time. Therefore, in order to accelerate the simulation speed, the ideal devices were used for fitting.

2. The parameters in the configuration file are designed based on actual IGCT data and should not be modified unless necessary.

**2. Recovery-enhanced Commutation Principle**

**Model composition:** As shown in Fig. 3 of the manuscript, the device-circuit coupling electromagnetic simulation model includes an equivalent DC source, a hybrid bridge, a thyristor bridge, and an equivalent AC source. The equivalent sources adopt LC oscillation to fit the voltage and current in the actual circuit, and the IGCT in the hybrid bridge adopts a physics-based compact model.

**Method of application:**

1. Before conducting simulation research, run the "Simulation_Recovery.m" configuration file to complete the parameter definitions in MATLAB.

2. Set the external circuit parameters in "Simulation_Recovery.slx", including all capacitors and inductors, as well as the snubber parameters of the devices.

3. Run the "Simulation_Recovery.slx" model to obtain the desired simulation results in SIMULINK.

**Points for attention:**

1. The recovery characteristics of thyristors were fitted using a simple circuit in the model.

2. The parameters in the configuration file are designed based on actual IGCT data and should not be modified unless necessary.
